# Use of statins and the risk of dementia and mild cognitive impairment: A systematic review and meta-analysis

**DOI:** 10.1038/s41598-018-24248-8

**Published:** 2018-04-11

**Authors:** Che-Sheng Chu, Ping-Tao Tseng, Brendon Stubbs, Tien-Yu Chen, Chia-Hung Tang, Dian-Jeng Li, Wei-Cheng Yang, Yen-Wen Chen, Ching-Kuan Wu, Nicola Veronese, Andre F. Carvalho, Brisa S. Fernandes, Nathan Herrmann, Pao-Yen Lin

**Affiliations:** 1 Department of Psychiatry, Kaohsiung Veterans General Hospital, Kaohsiung, Taiwan; 2 Center for Geriatric and Gerontology, Kaohsiung Veterans General Hospital, Kaohsiung, Taiwan; 3 Department of Psychiatry, Tsyr-Huey Mental Hospital, Kaohsiung Jen-Ai’s Home, Kaohsiung, Taiwan; 4 WinShine Clinics in Specialty of Psychiatry, Kaohsiung, Taiwan; 5 Physiotherapy Department, South London and Maudsley NHS Foundation Trust, London, UK; 6 Health Service and Population Research Department, Institute of Psychiatry, Psychology and Neuroscience (IoPPN), King’s College London, De Crespigny Park, London, UK; 7 Faculty of Health, Social Care and Education, Anglia Ruskin University, Chelmsford, UK; 8 Department of Psychiatry, Tri-Service General Hospital; School of Medicine, National Defense Medical Center, Taipei, Taiwan; 9 Institute of Brain Science, National Yang-Ming University, Taipei, Taiwan; 10 Department of Psychiatry, Tainan Hospital, Ministry of Health and Welfare, Tainan, Taiwan; 11 Department of Addiction Science, Kaohsiung Municipal Kai-Syuan Psychiatric Hospital, Kaohsiung, Taiwan; 12 Graduate institute of Medicine, College of Medicine, Kaohsiung Medical University, Kaohsiung, Taiwan; 13 Department of Adult Psychiatry, Kaohsiung Municipal Kai-Syuan Psychiatric Hospital, Kaohsiung, Taiwan; 14 Prospect clinic for otorhinolaryngology & neurology, Kaohsiung, Taiwan; 15 National Research Council, Neuroscience Institute, Aging Branch, Padova, Italy; 16 Department of Psychiatry, University of Toronto, Toronto, ON Canada; 17 Centre for Addiction & Mental Health (CAMH), Toronto, ON Canada; 18 IMPACT Strategic Research Centre, School of Medicine, and Barwon Health, Deakin University, Geelong, Australia; 19 Laboratory of Calcium Binding Proteins in the Central Nervous System, Department of Biochemistry, Federal University of Rio Grande do Sul, Porto, Alegre, Brazil; 20 Neuropsychopharmacology Research Group, Sunnybrook Health Sciences Centre, Toronto, ON, Canada; Department of Psychiatry, University of Toronto, Toronto, ON Canada; 21 Department of Psychiatry, Kaohsiung Chang Gung Memorial Hospital and Chang Gung University College of Medicine, Kaohsiung, Taiwan; 22 Institute for Translational Research in Biomedical Sciences, Kaohsiung Chang Gung Memorial Hospital, Kaohsiung, Taiwan

## Abstract

We conducted a systematic review and meta-analysis to investigate whether the use of statins could be associated with the risk of all-caused dementia, Alzheimer’s disease (AD), vascular dementia (VaD), and mild cognitive impairment (MCI). Major electronic databases were searched until December 27^th^, 2017 for studies investigating use of statins and incident cognitive decline in adults. Random-effects meta-analyses calculating relative risks (RRs) were conducted to synthesize effect sizes of individual studies. Twenty-five studies met eligibility criteria. Use of statins was significantly associated with a reduced risk of all-caused dementia (k = 16 studies, adjusted RR (aRR) = 0.849, 95% CI = 0.787–0.916, p = 0.000), AD (k = 14, aRR = 0.719, 95% CI = 0.576–0.899, p = 0.004), and MCI (k = 6, aRR = 0.737, 95% CI = 0.556–0.976, p = 0.033), but no meaningful effects on incident VaD (k = 3, aRR = 1.012, 95% CI = 0.620–1.652, p = 0.961). Subgroup analysis suggested that hydrophilic statins were associated with reduced risk of all-caused dementia (aRR = 0.877; CI = 0.818–0.940; p = 0.000) and possibly lower AD risk (aRR = 0.619; CI = 0.383–1.000; p = 0.050). Lipophilic statins were associated with reduced risk of AD (aRR = 0.639; CI = 0.449–0.908; p = 0.013) but not all-caused dementia (aRR = 0.738; CI = 0.475–1.146; p = 0.176). In conclusion, our meta-analysis suggests that the use of statins may reduce the risk of all-type dementia, AD, and MCI, but not of incident VaD.

## Introduction

As the life expectancy is getting longer worldwide, the number of people affected by cognitive decline and dementia is steadily increasing^1^. Dementia is an age-related neurodegenerative disease, characterized by a progressive decline in cognitive function often encompassing several domains (e.g. memory, attention, language, and problem solving)^2^. According to the World Health Organization (WHO) estimates, the number of individuals affected by dementia is expected to triple by 2050, with the rapid aging of populations globally [http://www.who.int/ageing/en/]. Meanwhile, the individual, societal and healthcare costs associated with dementia are steeply increased^3^. Unsurprisingly, the prevention of cognitive decline and dementia is a worldwide public health issue^4^.

Recently, interest has arisen in the potential for statins to delay cognitive decline in people with older age. Statins are drugs that inhibit 3-hydroxy-3-methylglutaryl coenzyme A (HMG-CoA) reductase^5^. Statins not only lower serum cholesterol levels, but also inhibit pivotal enzymatic reactions (e.g. the isoprenylation of a subset of GTPases)^6^ that lead to amyloid deposition and plaque formation; both are considered cornerstone pathways underpinning the development of Alzheimer’s disease (AD)^7^. Epidemiological data have supported a possible association between the use of statins and the risk of dementia, but evidence appears controversial. Some observational studies have demonstrated that statins reduce the risk of dementia or incident AD^8–17^, whereas several others have failed to replicate those findings^18–25^. Several possible factors may contribute to discrepant findings across studies. First, statins might exhibit neuroprotective effects limited only to earliest stages of AD^8,26^. Some studies have shown that statins exert more robust protective effects upon cognition for subjects younger than 80 at baseline^13,14^. Second, the degree of exposure (i.e. time and dose of statins use) could influence outcomes^14,15^. Third, clinicians might not be willing to prescribe statins for patients with pre-existing poor cognitive status due to concerns about possible treatment-related side effects, and lesser benefit because of expected diminished life expectancy^27,28^. Fourth, ApoE genotype might alter the association between use of statins and incidence of dementia. One recent study has shown that statins may be more beneficial in AD patients with homozygous ApoE4 genotypes^29^. Finally, the effects of confounders may limit inferences from observational studies^30^, and hence may vary depending on both the specific population being examined and potential confounding variables that are controlled for in multivariable analysis.

Several meta-analyses of observational studies have previously been conducted to examine the potential role of statins on incident mild cognitive impairment (MCI) or dementia, but have provided conflicting results thus far^31–36^. A meta-analysis including case-control and cohort studies demonstrated statins use did not confer a protective effect on the risk of dementia or AD^35^. However, another meta-analysis of 8 prospective cohort studies found that the use of statins was associated with a significantly reduced relative risks (RRs) of dementia by 39%^32^. In the most recent study published in 2013, Richardson *et al*. found a reduced RR for all-caused dementia, AD, or MCI based on observational studies, while 16 cohort studies were available when this previous meta-analysis was conducted^37^. The majority of these meta-analyses have focused solely on statins use and the risk of all-caused dementia and AD. Only one meta-analysis had examined a possible effect in reducing the incidence of MCI^37^ and to our knowledge, no previous meta-analyses has separately assessed the risk of vascular dementia (VaD). Several new reports (including new studies which included different (i.e. Asian) populations^38,39^ have been published in more recent years^36,38–41^. In addition, possibly due tolimited evidence no previous meta-analysis has explored potential sources of heterogeneity across studies.

Therefore, the current systematic review and meta-analysis aims to reappraise available evidence from prospective studies, which investigated whether statins use could diminish incident all-caused dementia, AD, VaD, and MCI. In addition, due to the anticipated larger current evidence base, we aimed to explore potential sources of heterogeneity across studies.

## Results

### Studies Included in the Meta-analysis

**Figure 1:**
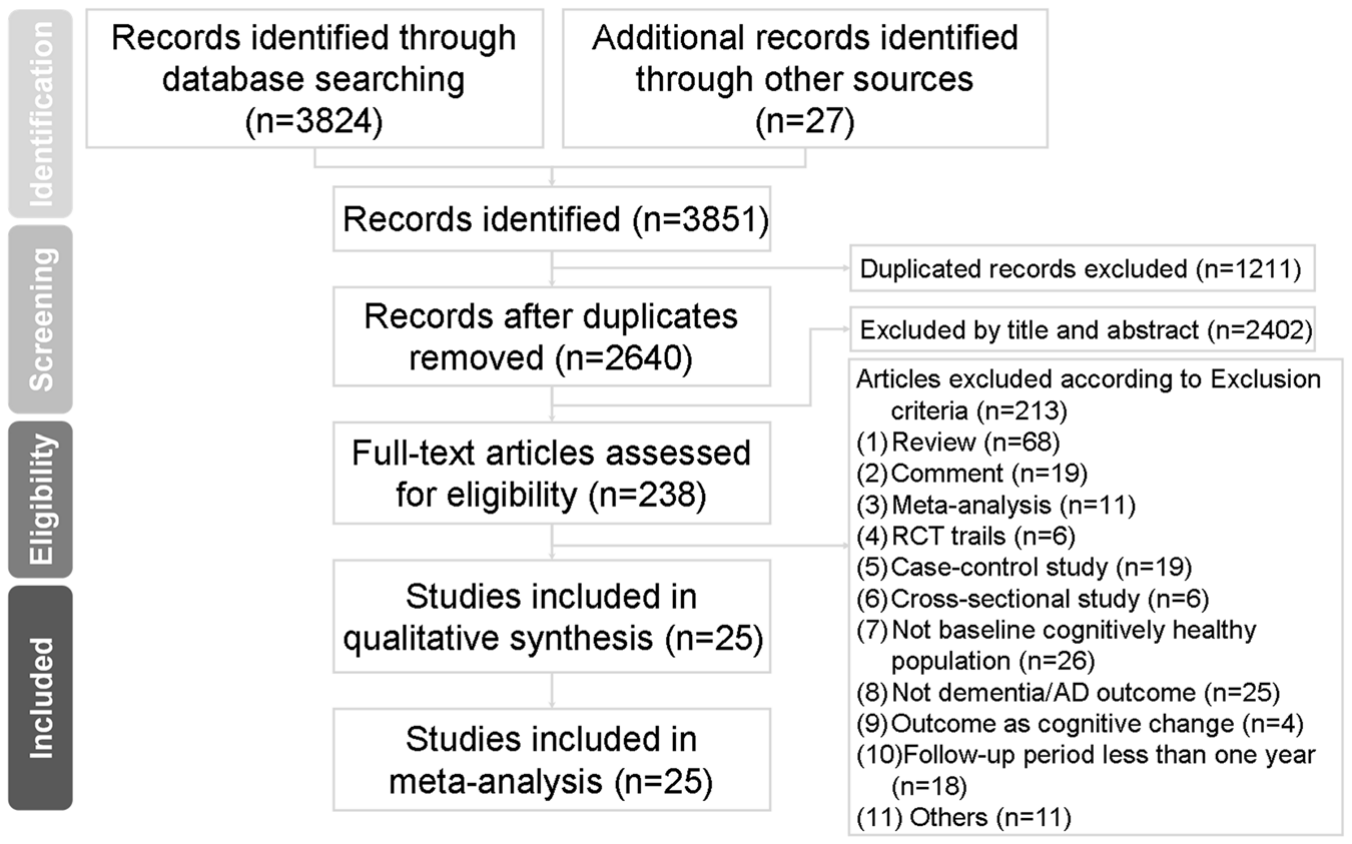
PRISMA flowchart of study selection for the current systematic review and meta-analysis.

Overall, 3,824 unique references were identified after database searching, while 27 were identified from other sources. Of those, 2402 were excluded after title/abstract screening because they are not related to this meta-analysis; the main target of current meta-analysis aimed to discuss the association between statins and dementia. Total 238 articles were scrutinized, and 213 were excluded with reasons (see Supplementary material Tables S1 and S2). Finally, 25 articles met inclusion criteria. The flow chart of the search strategy and results is depicted in Fig. 1. The mean total numbers of covariates adjusted were 7.40 (Standard deviation [SD]: 3.38) and mean duration of follow-up periods were 6.95 (SD: 5.39) years.

In total, twenty-five articles met the inclusion criteria and were included in the current systematic review and meta-analysis (Table 1)^8–10,13,16,18,19,21–23,25,36,38,40–51^. Table 1 provides characteristics of included studies.

**Table 1:** Characteristics of included studies.

Author (year)	Criteria	Study design	Mean age (SD) Male (%)	Subjects (case by statins use or total)*	Follow up (years)	Outcome*	Definition of statins use	Confirm statins use	Dropouts (%)
Harding (2017)^35^	MCI^a^	Prospective cohort	69.7 0	175(SU/NSU: 3/14)	8.4	MCI	Any use	Self-reported	n/a
Chitnis (2015)^39^	ICD-9	Retrospective cohort	74.4 (9.2) 47	8062 (SU/NSU: 512/623)	3	Dementia	Current use >15 days during month	EMR^e^	n/a
Hendrie (2015)^40^	DSM-IV ICD-10	Prospective cohort	76.6 (4.9) 30.3	974 (Dementia, SU/NSU: 9/56); 965 (AD, SU/NSU: 7/49)	8	Dementia; AD	Any use	Medication inspection	n/a
Chen (2014)^37^	DSM-IV	Retrospective cohort	66.8 (8.6) 52.3	18170 (Dementia, SU/NSU: 53/824); 18013 (AD, SU/NSU: 41/679)	8	Dementia; AD	Regular use (more than one year)	EMR^f^	n/a
Ancelin (2012)^18^	DSM-IV NINCDS-ADRDA	Prospective cohort	74 40	6830 (Dementia, 483; AD, 332)	7	Dementia; AD	Use at baseline	Medication inspection	n/a
Bettermann (2012)^8^	n/a	Prospective cohort	78.6 (3.3) 54	2587 (Dementia, 324; AD, 212; VaD, 148)	6	Dementia; AD; VaD	Any use	Medication inspection	n/a
Beydoun (2011)^41^	DSM-III-R NINCDS-ADRDA Petersen criteria	Prospective cohort	58.0 (18) 62	1604 (Dementia, 259); 1308 (MCI, 133)	25	Dementia; AD; MCI	Ever use	Medication inspection	n/a
Parikh (2011)^43^	n/a	Retrospective cohort	75.5 (6.1) 98	377838 (SU/NSU: 5316/9246)	2	Dementia	Any use (through study)	Pharmacy records	n/a
Hippisley-Cox (2010)^42^	n/a	Prospective cohort	45.8 (14.1) 45	2004692 (8784)	Up to 6	Dementia	New users	EMR^g^	n/a
Li (2010)^13^	DSM-IV NINCDS-ADRDA	Prospective cohort	75.4 (6.2) 40.7	3099 (263)	6.1	AD	Any use (3 consecutive)	Pharmacy records	8%
Haag (2009)^10^	DSM-III-R NINCDS-ADRDA	Prospective cohort	69.4 (9.1) 40	6992 (466)	9	AD	Any use	Pharmacy records	n/a
Solomon (2009)^45^	n/a	Prospective cohort	71.8 (4.9) 46.3	14294 (1301)	20	Dementia	n/a	EMR^h^	n/a
Schneider (2009)^44^	MCI^b^	Prospective cohort	70 to 80 0	293 (n/a)	3	MCI	Continuous use (2 consecutive)	n/a	n/a
Arvanitakis (2008)^19^	n/a	Prospective cohort	74.9 (7.0) 31.3	929 (SU/NSU:: 16/175)	Up to 12	AD	Any use	Medication inspection	8%
Cramer (2008)^9^	DSM-IV NINCDS-ADRDA MCI^c^	Prospective cohort	70.4 (6.0) 42	1674 (Dementia, SU/NSU: 28/102; MCI, 130)	5	Dementia; MCI	Any use	Medication inspection	31%
Sparks (2008)^16^	ICD-9 NINCDS-ADRDA	Clinical trial cohort	74.8 (3.8) 46	2068 (SU/NSU: 4/20; MCI, n/a)	4	AD; MCI	Continuous use at all visits	Self-reported	12%
Li (2007)^50^	DSM-IV NINCDS-ADRDA	Prospective cohort	74.1 (3.8) 67.2	110 (12)	n/a	AD	Any use (3 consecutive)	Pharmacy records	n/a
Szwast (2007)^49^	DSM-III-R ICD-10	Prospective cohort	77.3 (5.3) 38.1	1141 (SU/NSU: 3/29)	3	Dementia	Use at baseline	Medication inspection	28%
Wolozin (2007)^48^	ICD-9	Prospective cohort	75 94	1290071 (SU/NSU: 3361/3359)	3	Dementia	Continuous use in first 7 months	EMR^i^	n/a
Zigman (2007)^46^	n/a	Prospective cohort	41 to 78 22.8	123 (SU/NSU: 8/30)	5.5	Dementia	Any use	Medication inspection	n/a
Rea (2005)^22^	NINCDS-ADRDA	Prospective cohort	75 40	2798 (Dementia, SU/NSU: 38/438; AD, SU/NSU: 21/216; VaD, SU/NSU: 7/55)	5	Dementia; AD; VaD	Any use	Medication inspection	n/a
Zandi (2005)^25^	DSM-III-R NINCDS-ADRDA	Cross-sectional and prospective cohort	75.5 (7.1) 42.8	3308 (Dementia, SU/NSU: 8/174; AD, SU/NSU: 4/98)	3	Dementia; AD	Any use	Medication inspection	27%
Li (2004)^21^	DSM-IV NINCDS-ADRDA	Prospective cohort	75.1 (6.1) 40.2	2356 (Dementia, SU/NSU: 41/271; AD, 168)	6	Dementia; AD	Any use (2 consecutive within 6 months)	Pharmacy records	9%
Reitz (2004)^23^	NINCDS-ADRDA	Cross-sectional and prospective cohort	78.4 (6.2) 31.7	2126 (AD, 119; VaD, 54)	4.8 ± 2.9	AD; VaD	Any use	Medication inspection	45.1%
Yaffe (2002)^47^	MCI^d^	Clinical trial cohort	<80 0	2126 (SU/NSU: 37/42)	4	MCI	Current use	Medication inspection	n/a

^a^Defined as decline in Consortium to Establish a Registry for Alzheimer’s Disease (CERAD) word list.

^b^Defined by Trails-A and B, HVLT-immediate and delayed recall.

^c^Defined as decline in 3MS, SEVLT.

^d^Defined by level of 3 MS examination.

Note: the datasets included in this meta-analysis were extracted from the peer-reviewed articles which are not available from open access sources.

Name of the EMR of each study: ^e^Medicare Advantage Prescription Drug plan [MAPD] in Texas; ^f^National Health Insurance Research Database in Taiwan; ^g^Egton Medical Information System [EMIS] in England and Wales; ^h^Hospital Discharge Registry and Drug Reimbursement Registry in Finland; ^i^US Veterans Affairs database.

Abbreviation: Abbreviations: 3MS: Modified mini-mental state examination; AD = Alzheimer’s Disease; ApoE = Apolipoprotein E; BMI = Body Mass Index; CABG = Coronary artery bypass graft; CAD = Cardiovascular disease; CASI = Cognitive ability screening instrument; CCI = Charlson comorbidity index; CHD = Coronary Heart Disease; DM = Diabetes; EMR = Electronic Medical Records; HTN = Hypertension; HVLT = Hopkins Verbal Learning Test; LDL-C = Low-density lipoprotein cholesterol; LLA = Lipid lowering agents; MCI = Mild cognitive impairment; MR = Mental retardation; N/A = Not Applicable; NSU: No Statin Use; OHA = Oral hypoglycemic agents; SEVLT: Spanish and English Verbal Learning Test; SSRI = Selective serotonin reuptake inhibitor; SU: Statin User; TCA = tricylic antidepressants.

Among included studies, 16 studies investigated the association of statins use and incident all-caused dementia (N = 2,745,149, mean age 59.3 years, male = 67.3%, incident cases_ = _35,688), 14 studies assessed the association of statins use and incident AD (N = 52,218, mean age 71.3 years, male = 45.3%, incident cases = 3120), 3 studies investigated VaD (N = 5,987, mean age 77.2 years, male = 42.5%, incident cases = 422), and 6 studies investigated MCI (N = 6,808, mean age 68.4 years, male = 33.2%, incident cases of four studies^9,36,42,48^ = 359), respectively. Across those 25 studies, Supplementary material Table S3 provides a list of potential confounders considered in the multivariable models in each study, and the total number of covariates considered in the most fully adjusted risk estimate (e.g. aRRs) in each included study.

### Methodological Quality Assessment

The average NOS were 7.72 with interquartile range (IQR) (7.00–8.00). The score in each domain of the NOS is provided in Supplementary material Table S4.

### Statins and Incident All-caused Dementia

**Figure 2:**
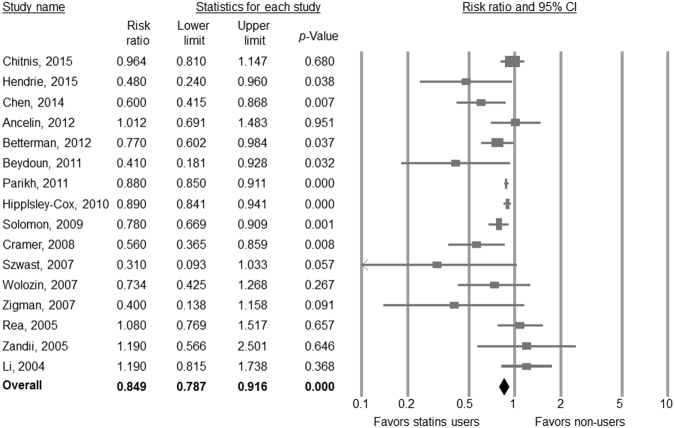
Forest plot of random-effects meta-analyses of the use of statins and incidence of all-caused dementia.

Across 16 studies^8,9,18,21,22,25,38,40–44,46,47,49,50^, participants who received statins were significantly less likely to develop all-caused dementia compared to those who were not treated with statins (aRR from 16 studies = 0.849, 95% CI = 0.787–0.916, p < 0.001) (Fig. 2). There was no evidence of publication bias according to Egger’s test (t value = 1.816, df = 14, p = 0.091). However, heterogeneity was verified (Q value = 29.778, df = 15, I^2^ = 49.627, p = 0.013).

### Sources of Heterogeneity

**Figure 3:**
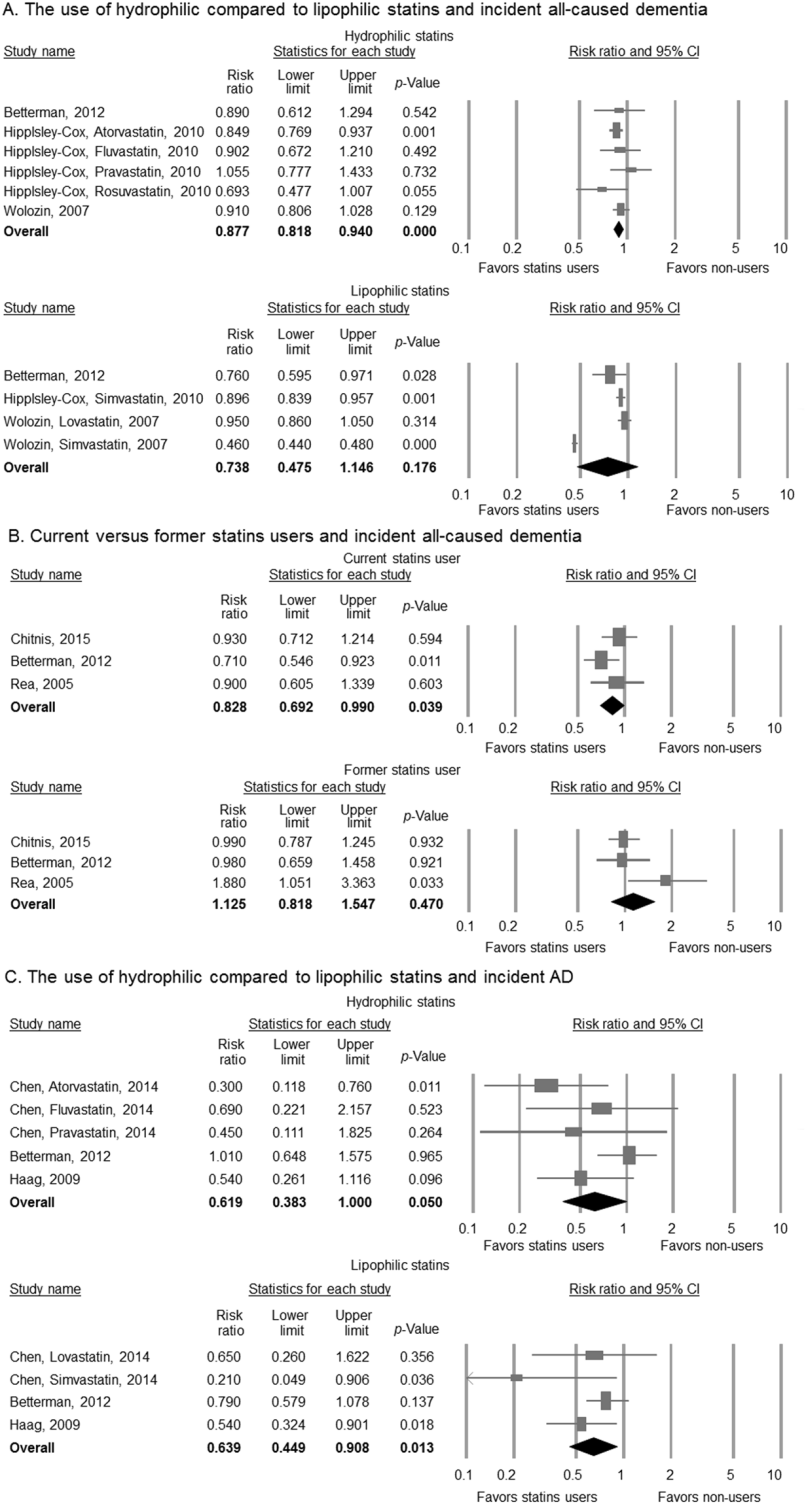
Subgroup analyses. (**A**) The use of hydrophilic compared to lipophilic statins and incident all-caused dementia; (**B**) current versus former statins users and incident all-caused dementia; and (**C**)The use of hydrophilic compared to lipophilic statins and incident Alzheimer’s Disease. Squares depict individual studies and diamonds depict summary effect size estimates (aRRs).

Subgroup analysis showed the use of hydrophilic statins were associated reduced risk of all-caused dementia (aRRs = 0.877; CI = 0.818–0.940; p < 0.001), whereas the use of lipophilic statins were not associated with this outcome (aRR = 0.738; CI = 0.475–1.146; p = 0.176) (Fig. 3A). Regarding status of statins use (current and former users), lower risk of all-caused dementia was found among current users (aRR = 0.828; CI = 0.692–0.990; p = 0.039) but not in former users (aRR = 1.125, CI = 0.818–1.547, p = 0.470) (Fig. 3B). In the meta-regression analyses, mean age, percentage of male, education in years, study duration, percentage of cardiovascular, cerebrovascular disease, DM, HTN, smoking, ApoE4 status, BMI > 25, cholesterol > 200 mg/dl, total Newcastle scores, and total number of covariates used in the multivariate analyses did not seem to contribute to heterogeneity. The percentage of white population (slope = 0.005; p = 0.006) and most notably the percentage of cholesterol > 200 mg/dl (slope = 0.018; p = 0.009) emerged as significant moderators of this outcome (Supplementary material Table S5).

### Statins and Incident Alzheimer’s Disease

**Figure 4:**
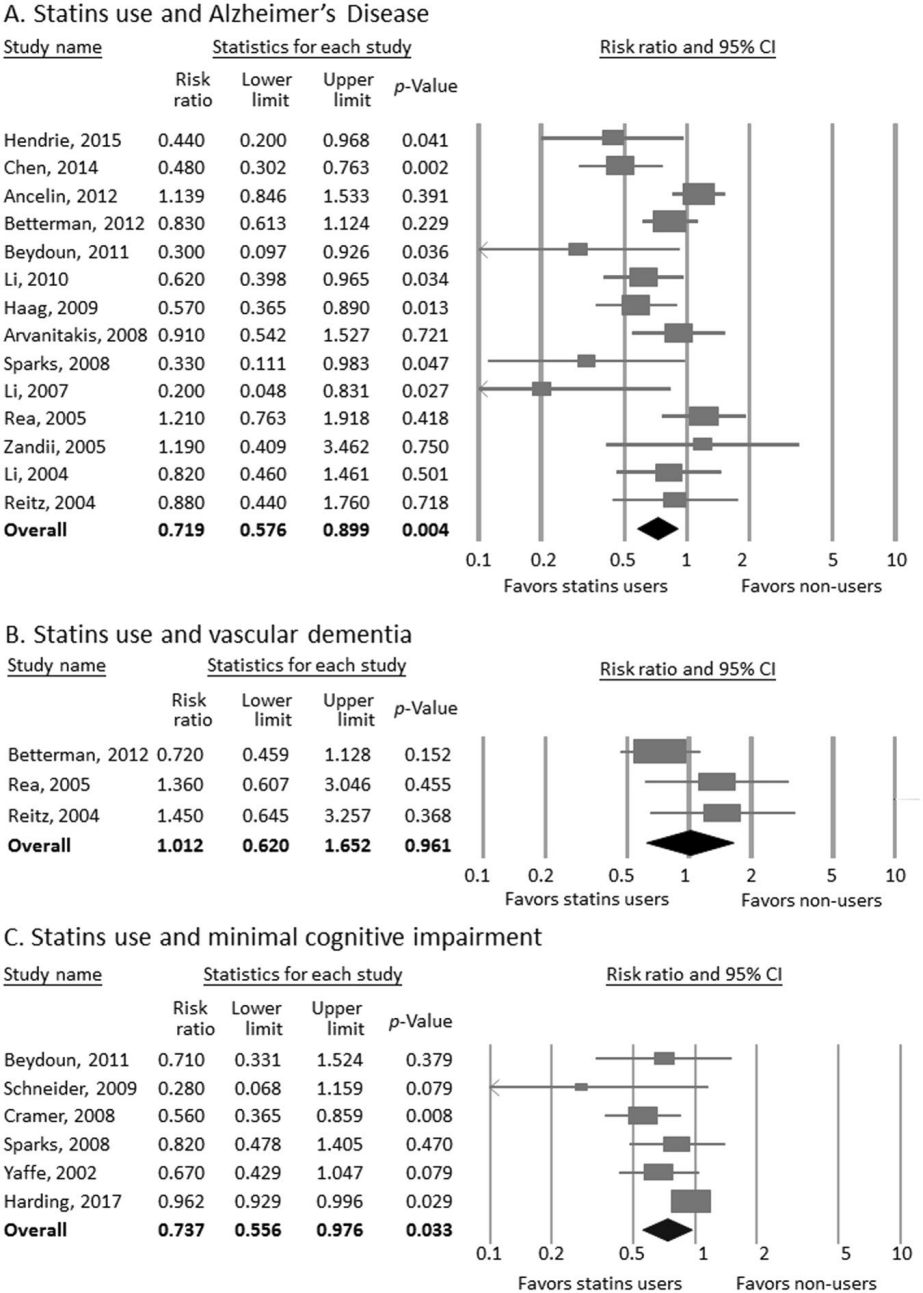
Forest plot of random-effects meta-analyses of the use of statins and incidence of (**A**) Alzheimer’s Disease, (**B**) Vascular dementia, and (**C**) Mild cognitive impairment. Squares depict individual studies and diamonds depict pooled effect sizes (aRRs).

Across 14 studies^8,10,13,16,18,19,21–23,25,38,41,42,51^, participants who were treated with statins were significantly less likely to develop AD compared to those who were not treated with statins (aRR from 14 studies = 0.719, 95% CI = 0.576–0.899, p = 0.004). There was evidence of publication bias through Egger’s test was observed (t value = 2.307, df = 12, p = 0.039) (Fig. 4A). We used Duval and Tweedie’s trim and fill procedure toward the right to adjust effect size estimates to publication bias and the results remained significant (aRR = 0.814, 95% CI = 0.713–0.930). In addition, heterogeneity was large (Q value = 28.779, df = 13, I^2^ = 54.828, p = 0.007). Therefore, potential sources of heterogeneity were explored.

### Sources of Heterogeneity

Subgroup analyses showed that the use of lipophilic statins was associated reduced risk of AD (aRR = 0.639; CI = 0.449–0.908; p = 0.013), while the use of hydrophilic statins reduced the risk of incident AD at the statistical trend level (aRRs = 0.619; CI = 0.383–1.000; p = 0.050) (Fig. 3C). We could not perform the association between statins use (current and former users) and the risk of AD because of recruited studies less than 3. In meta-regression analyses, the percentage of white participants (slope = 0.006; p = 0.047), study duration (slope = −0.063; p = 0.033), and percentage of apoE4 > or = 1 (apoE4 carriers) (slope = −0.042; p = 0.044) emerged as significant moderators of outcomes (Supplementary material Table S5).

### Statins use and Incident Vascular Dementia

Across 3 studies^8,22,23^, no significant difference in the incidence of VaD was observed between participants with statins treatment and those without (aRR = 1.012, 95% CI = 0.620–1.652, p = 0.961). There was evidence of publication bias through Egger’s regression (t value = 17.932, df = 1, p = 0.035). We used Duval and Tweedie’s trim and fill procedure toward left to adjust effect size estimates to publication bias and the results remained non-significant (aRR = 0.720, 95% CI = 0.428–1.210). No significant heterogeneity was found (Q value = 3.256, df = 2, I^2^ = 38.581, p = 0.196) (Fig. 4B).

### Statin use and Incident MCI

Across 6 studies^9,16,36,42,45,48^, participants who were using statins were significantly less likely to develop MCI compared with those who did not receive statins (aRR = 0.737, 95% CI = 0.556–0.976, p = 0.033). There was evidence of publication bias as indicated by Egger’s regression (t value = 4.051, df = 4, p = 0.015). We used Duval and Tweedie’s trim and fill procedure toward the right to adjust effect size estimates to publication bias and the results tend to insignificance (aRR = 0.923, 95% CI = 0.713–1.193). Additionally, there was significant heterogeneity was found (Q value = 12.330, df = 5, I^2^ = 59.449, p = 0.031). Therefore, potential sources of heterogeneity were explored.

### Sources of Heterogeneity

In meta-regression analyses, the percentage of male proportion (slope = −0.008; p = 0.022) and number of covariables (slope = −0.114; p = 0.011) emerged as significant moderators of outcomes (Supplementary material Table S5).

### Adverse Events

None of studies, except one^43^, reported information regarding side effects during follow-up period. Hippisley-Cox *et al*.^43^ reported that the use several statins including simvastatin, atrovastatin, fluvastatin, pravastatin, and rosuvastatin increased the risk of liver dysfunction (simvastatin 10–20 mg/day, for women, adjusted HR [aHR] = 1.47, 95% CI = 1.32–1.63, for men, aHR = 1.35, 95% CI = 1.25–1.54), myopathy (simvastatin 10–20 mg/day, for women, aHR = 2.91, 95% CI = 2.19–3.88, for men, aHR = 6.12, 95% CI = 4.97–7.55), acute renal failure (simvastatin 10–20 mg/day, for women, aHR = 1.38, 95% CI = 1.10–1.74, for men, aHRs = 1.39, 95% CI = 1.14–1.70), and cataract (simvastatin 10–20 mg/day, for women, aHR = 1.30, 95% CI = 1.24–1.36, for men, aHR = 1.32, 95% CI = 1.24–1.39).

## Discussion

The current meta-analysis of 25 cohort studies provides the most comprehensive meta-analytic evidence regarding the effects of statins on incident all-caused dementia, AD, VaD, and MCI. We found that statins users, without baseline cognitive dysfunction, had a significantly reduced risk of developing all-caused dementia, AD, and MCI. Statins use was associated with a 15.1%, 28.1%, and 26.3% lowered risk of developing all-caused dementia, AD, and MCI respectively. Nevertheless, the few studies available provided no evidence that statins use could prevent the onset of VaD. In addition, the larger body of data included in the current meta-analysis compared to previous similar efforts^37,52^ allowed a more accurate exploration of potential sources of heterogeneity.

The current meta-analysis suggests that statins may offer a more significant preventative benefit for neurodegenerative dementing illness such as AD than for all-caused dementia^31–34,37,53^. Besides, both our study (aRR = 0.737, 95% CI = 0.556–0.976) and previous work by Richardson *et al*. found the statins appear to provide the stronger protective effects for incident MCI (aRR = 0.66, 95% CI: 0.51–0.86). However, compared to the Richardson study, we added two studies and enrolled more subjects in the present meta-analysis^36,45^. Nonetheless, the evidence base appears to consistently indicate that statins may protect against early stage cognitive decline, such as MCI^8,9,36^. A longitudinal study of 6,600 subjects with normal cognition or MCI even showed statins exert a cognitive protection among those with normal cognition at baseline but not MCI, indicating statins might exert benefit before MCI status^54^.

Importantly, this is the first meta-analysis to examine the association between statins use and risk of VaD, although no significant differences emerged (aRR = 1.012, 95% CI = 0.620–1.652, p = 0.961). Atherosclerosis is considered to be responsible for diffuse periventricular white matter abnormalities, which are one of major pathophysiological mechanisms of VaD^55^. We hypothesized that statins may reduce the development of VaD via alleviating or preventing cerebrovascular disease, which are the main risk factors for VaD^56–58^. However, the negative findings between VaD and statins from this meta-analysis might be due to the few number of included studies. Nevertheless, we considered adjust relative risks of individual studies. Therefore, it is possible that statins might not offer an independent preventative benefit for VaD. Besides, these studies did not account for important factors (such as plasma lipids or apolipoprotein E genotype)^8,22,23^, which might influence the results. Thus, clearly more research is required to consider if statins may confer a protective effect over future VaD.

The exact mechanism of statins on cognitive protection in older adults has not been elucidated but it might include pleiotropic effects on several mechanistic pathways. First, in animal models, statins could decrease cholesterol levels and attenuate formation of beta-amyloid (β-amyloid), which is main component of amyloid plaques in the brains of individuals with AD^59^. Besides, statins in preclinical models reduced brain cholesterol levels, leading to lower neurofibrillary tangles^60^. These effects could result in lower risk of dementia. The meta-regression analysis of present study could support this hypothesis, showing statins provide higher cognitive protective effect in studies with higher percentage of cholesterol >200 mg/dl (slope = 0.018, p = 0.009). Second, statins may exert anti-inflammatory effects in the brain. For example, statins significantly reduced β-amyloid-induced production of pro-inflammatory cytokines, such as interleukin (IL)-1beta, IL-6 and tumor necrosis factors-gamma (TNF-gamma) in the hippocampus^61^. This could be a relevant effect since accumulating evidence indicating that both peripheral immune activation and neuroinflammation are involved in the pathophysiology of AD. Third, statins exert cholesterol-independent effects (also referred to as pleiotropic effects), that showed antiproliferative and antithrombotic benefits and improved endothelial dysfunction, which plays import role in the initiation of inflammatory process-atherosclerosis^62^. Taken together, statins could attenuate the cognitive decline via, at least in partial, decreasing anti-inflammatory action, attenuate formation of beta-amyloid (β-amyloid) and neurofibrillary by lowering cholesterol levels, and pleiotropic effects.

Our subgroup analyses show that significant reduced risks of all-caused dementia and marginal significances of lower risk of AD were found for hydrophilic statins. In addition, lipophilic statins could reduce risks of AD but not all-caused dementia. The protective effects of statins on preventing the onset of dementia which can emerge as a function of their pharmacokinetic properties remains under debate, with some studies favoring lipophilic statins^38^ but other showing no difference between hydrophilic and lipophilic statins^10^. In the current meta-analysis, hydrophilic and lipophilic statins significantly decreased the incidence of all-caused dementia and AD, respectively. Studies had shown the lipophilic statins can more easily than hydrophilic statins cross the blood-brain barrier, thus potentially providing more robust benefits for the prevention of neurodegenerative disease^63,64^. Our results suggest that hydrophilic statins could exhibit more extensive protective effects than lipophilic statins in preventing all-caused dementia and possibly AD. However, these results should be interpreted cautiously. It is noteworthy that both forest plots of hydrophilic and lipophilic statins tended to the left, which means favor statins‘ preventive effect on all-caused dementia and AD. Although some results such as association between lipophilic statins and all-caused dementia and between hydrophilic statins and AD did not show significant differences, these make us not to reject the null hypothesis. Additionally, two recent epidemiological studies without non-statins comparator groups and therefore did not meet eligibility criteria for this study warrant mentioning. Zissimopoulos *et al*. used administrative claims data from Medicare beneficiaries to analyze the association between statin use with AD onset. They found lipophilic (simvastatin and atorvastatin) and hydrophilic (pravastatin and rosuvastatin) generally were associated with reduced risk of AD depend on different races and sex^65^. In contrast, Sinyavskaya *et al*. reported lipophic statins (simvastain, atorvastatin, fluvastatin) were not associated with decreased incident of AD compared to hydrophilic statins (pravastain, rosuvastatin), using data from the UK Clinical Practice Research Datalink cohort of patients aged 60 to 95 years who were followed for a median of 5.9 years^66^. Therefore, the association of statins and AD and all-caused dementia might vary across different properties of statins, sex, and race/ethnicity. Future intervention studies are needed to better study the differential effects of hydrophilic and lipophilic statins on preventing incidence of dementia or otherwise as novel neuroprotector agents for MCI and AD.

The meta-regression analysis showed several variables were associated with statins use and the relationship with the risk of all-caused dementia, AD and MCI. One of interesting findings was statins expressed lesser cognitive preventive effect in studies with higher percentage of white ethnicity in both all-caused dementia (slope = 0.005, p = 0.006) and AD (slope = 0.006, p = 0.047). Pharmacokinetic differences exist among different races^67^. Caucasian subjects tend to have lower plasma exposure to and its metabolites compared non-Caucasian subjects in several types of statins^68,69^, leading to lesser treatment effect. Nearly all of recruited studies are from Western countries. The sample sizes for other ethnicities are smaller, and the estimates might be less precisely measured. Future studies to address neuroprotective effects of statins in difference ethnicities are needed. In addition, apoE4 allele is a major genetic risk factor for both all-caused dementia and AD^70^. It is reasonable that apoE might alter the effects of statin on the cognition as apoE plays a critical role in the transport of cholesterol and fats to the brain^71^. Indeed, we did find significant negative association between presence of apoE4 and AD (slope = −0.042, p = 0.044), although not in all-caused dementia.

Some important cofounders should be considered. For example, studies have shown that different levels of social relationship^72^ and physical exercise^73^ might relate to the development of dementia. To determine the intensity of social involvement is challenge and lack of objective measures in the past. Currently, there is an increasing number of commercially available devices (wearable activity trackers), which provide researchers with secure and precise means to collect, and analysis data generated by health devices^74^. This wearable technology could be integrated into the future cohort studies to off real-time monitoring to improve dementia prevention and more person-centered care.

Several limitations should be addressed. First, the study is a pooled synthesis of observational studies which are susceptible to the effects of chance, bias and confounding and thus, results should be interpreted cautiously. Second, we excluded studies that considered cognitive function change as endpoint and did not discuss the clinical trajectory at every clinical assessment point until the last assessment. However, this is beyond the scope of current meta-analysis. Although the U.S. Food and Drug Administration (FDA) issued a statement regarding poor cognitive status associated with statin use in 2012, no convincing evidence so far support this warning^75,76^. Nevertheless, it would be potentially more informative to analysis all studies with investigating the effect of statins on the cognitive function change measured by objective tools. Future studies could be conducted to address this issue. Third, we only included peer-reviewed articles published in English language and thus it is possible that we might have missed some non-English studies. However, no publication bias was evident for almost all our outcomes. Fourth, meta-regression could not be performed for studies of VaD and MCI due to few datasets available. Fifth, none of the included studies used a propensity score or a matching criterion between statins users or not and so another potential bias could be present in our findings. In an attempt, to provide a better control of potential confounders we also performed meta-analyses considering the most fully adjusted outcome measures of individual studies. Finally, several diagnostic criteria for dementia (DSM, ICD, and NINCDS-ADRDA etc.) as well as different methods to assess the use of statins (electronic medical records, pharmacy records, and medication inspection etc.) across included studies may also limit the comparability and synthesis of studies included in this meta-analytic review.

## Conclusions

The use of statins appears to have beneficial effects in reducing incidence of all-caused dementia, AD, and MCI, but not VaD. Subgroup analyses suggest that hydrophilic and lipophilic statins showed a beneficial effect in preventing all-caused dementia and AD, respectively. Thus, our data suggest that the use of statins may confer a potential benefit for the prevention of dementia.

## Methods and Materials

The current systematic review and meta-analysis was in line with the Preferred Reporting Items for Systematic Reviews and Meta-Analyses (PRISMA) guidelines^77^ (Supplementary material Table S6). This systematic review and meta-analysis was approved by the Institutional Review Board of the Tri-Service General Hospital (TSGHIRB: B-105-12). Two investigators (CSC and PTT) have independently performed database searching, study selection, data extraction, and the rating of the methodological quality of included studies. Disagreements were resolved through consensus.

### Database Searching

The PubMed, ScienceDirect, Psychology and Behavior Sciences Collection, ClinicalTrials.gov and Cochrane library were systematically searched from inception up until December 27^th^, 2017 (Supplementary material Table S7). The search string used for the electronic database search was provided in the Supplementary material. The search strategy was augmented through hand searching the reference lists of included articles, as well as previous reviews^52,76^ and meta-analysis^37^ on the topic.

### Eligibility Criteria and Study Selection

The following eligibility criteria were applied: (1) prospective cohort studies investigating the effects of statins vs. participants not taking statins; (2) participants had to be cognitively healthy at baseline and without a history of cognitive dysfunction; (3) outcome measures had to include either incident all-caused dementia or otherwise specific-caused dementia (i.e. AD or VaD) or mild cognitive impairment (MCI) based on Diagnostic and Statistical Manual of Mental Disorders (DSM)^2^, International Classification of Diseases (ICD)^78^, National Institute of Neurological and Communicative Disorders and Stroke and the Alzheimer’s Disease and Related Disorders Association (NINCDS-ADRDA)^79^, diagnosis of MCI was based on Petersen’s, International Working Group, or National Institute on Aging-Alzheimer’s Association workgroups diagnostic systems^80,81^; (4) with a follow-up period longer than one year and (5) peer-reviewed article written in English language.

The exclusion criteria were: (1) cross-sectional studies; (2) retrospective case-control studies; (3) clinical trials (i.e. randomized controlled trial (RCT)); and (4) studies that did not assess incident dementia or MCI at endpoint; (5) conference abstracts and studies published in languages other than English.

### Methodological Quality Assessment

We used the Newcastle-Ottawa Scale (NOS) to rate the methodological quality of included studies^82^. For prospective cohort studies, the NOS considers three domains: selection of participants (maximum of four stars), comparability of groups (maximum of two stars), and measurement (maximum of three stars). Scores were ranged from 0 (the lowest) to 9 (the highest), and a score higher or equal to 7 indicated high methodological quality.

### Data Extraction

The primary outcomes were risk of all-caused dementia, AD, VaD, and MCI considering statins use as the exposure. Pooled estimates between statins use and dementia were calculated by using fully adjusted RRs (aRRs) provide in each publication (further details in meta-analysis section).

A pre-specified data extraction form was used to extract data for this meta-analysis. Data was extracted for each study included basic characteristics of participants (age, percentage of male, education in years, percentage of whites), study duration (follow-up time, in years), substance use (percentage of alcohol and smoking), prevalence of co-occurring medical conditions (cardiovascular, cerebrovascular, diabetes mellitus [DM], hypertension), percentage of > or = 1 Apolipoprotein E epsilon 4 (ApoE4) (ApoE4 carrier), cholesterol > 200 mg/dl and percentage of overweight/obese people (BMI > 25, (%)). Diagnosis of all-caused dementia, AD, and VaD were based on DSM-III-R^10,25,42,50^, DSM-IV^9,13,18,21,38,39,41,51^, NINCDS-ADRDA^9,10,13,16,18,21–23,39,42,51^, ICD-9^16,40^, or ICD-10 ^41,49,50^; some studies did not provide the criteria for interested outcome^8,19,43,44,46,47^; diagnosis of MCI was defined based on Petersen criteria^42^, probable prodromal AD^16^ or decline of specific cognitive decline (e.g. modified mini-mental state examination, Verbal Learning Test and Consortium to Establish a Registry for Alzheimer’s Disease (CERAD) word list)^9,36,45,48^.

When data were not available in the articles, we electronically contacted the authors in at least two separate occasions to provide additional data.

### Meta-analysis

All meta-analytic procedures were performed with the Comprehensive Meta-Analysis software, version 3 (Biostat, Englewood, NJ) software. For each meta-analysis, we considered the risk measure of each study which was most fully adjusted for potential confounders. We conducted the meta-analyses with random-effects models due to the anticipated heterogeneity across studies. Heterogeneity was assessed with the Cochran Q test and the I^2^ metric^83^. We changed ORs to RRs if data available in three recruited studies^41,50,51^ using the formula suggested by Cochrane Hanbook^84^. Therefore, the effect size (ES) and its 95% confidence interval [CI] was defined as RRs to indicate the difference of the incident rate of all-caused dementia (including AD, VaD, or MCI) as a function of statins use (exposure). A similar analysis was undertaken pooling the RR of each study, adjusted for the highest number of potential confounders available.

Subgroup analyses stratified by pharmacokinetic properties of statins (hydrophilic vs lipophilic), as previous studies, showed inconsistent results regarding the protective effect of hydrophilic and lipophilic statins on cognition^10,38^. Atovastatin, fluvastatin, pravastatin, and rosuvastatin are hydrophilic statins, whereas cerivastatin, lovastatin, and simvastatin are more lipophilic statins^84,85^. Besides, status of statins usage (current and former use) was undertaken. Subgroup analyses were performed when data from at least three datasets.

Publication bias was assessed via a visual inspection of funnel plots and by means of the Egger’s regression test^86^. Additionally, to account for publication bias, we used the trim-and-fill method, based on the assumption that the effect sizes of all the studies were normally distributed around the center of a funnel plot; in the event of asymmetry, this method adjusts for the potential effect of unpublished (trimmed) studies^86^. In addition, we conducted a meta-regression analysis using an unrestricted maximum likelihood method to explore potential moderators. The following variables were considered for meta-regression analyses: mean age, male gender (%), education (years), percentage of ethnicity, study duration (follow-up duration, in years), and prevalence comorbidities (cardiovascular, cerebrovascular, DM, and hypertension), percentage of presence of ApoE4 (ApoE4 carriers), total NOS scores, BMI > 25, cholesterol > 200 mg/dl and numbers of covariates adjusted. The meta-regression procedure was only undertaken when moderator variables were available from more than 5 individual studies. Statistical significance was set as two-tailed P value less than 0.05.

## Electronic supplementary material

Supplementary Online Materials
