# Supplementary material for: Use of statins and the risk of dementia and mild cognitive impairment: A systematic review and meta-analysis
Source: Sci Rep. 2018 Apr 11;8:5804. doi: 10.1038/s41598-018-24248-8 (PMC5895617; doi:10.1038/s41598-018-24248-8)
Supplement: Supplementary file 1 — Supplementary Online Materials [file 41598_2018_24248_MOESM1_ESM.pdf]

Use of statins and the risk of dementia and mild cognitive impairment: A systematic review and meta-analysis

Che-Sheng Chu, Ping-Tao Tseng, Brendon Stubbs, Tien-Yu Chen, Chia-Hung Tang, Dian-Jeng Li, Wei-Cheng Yang, Yen-Wen Chen, Ching-Kuan Wu, Nicola Veronese, Andre F. Carvalho, Brisa S Fernandes, Nathan Herrmann, and Pao-Yen Lin

Supplementary Online Materials

Supplemental Table S1: Excluded studies with reasons.

Studies excluded: n = 213

(detail please see reference list in the supplementary material Table S2)

Review n = 68 (1-68)

Comment n = 19 (69-87)

Meta-analysis study n = 11 (88-98)

RCT trials (reason written in method) n = 6 (99-104)

Case-control study n = 19 (105-123)

Cross-sectional study n = 6 (124-129)

Not baseline cognitively healthy population n = 26 (130-155)

Not dementia/AD outcome n = 25 (156-180)

Outcome as cognitive change n = 4 (181-184)

Follow-up period less than one year n = 18 (185-202)

Others n = 11 (same population = 2 (203, 204); animal study = 2 (205, 206); quasi-experimental study = 1 (207); study protocol = 2 (208, 209); no data available = 2 (210, 211); no non-statin control group = 2 (212, 213)

Supplemental Table S2: supplementary references of recruited studies

1. Alegret M, Silvestre JS. Pleiotropic effects of statins and related pharmacological experimental approaches. Methods and findings in experimental and clinical pharmacology. 2006; 28(9): 627-56.

2. Algotsson A, Winblad B. Patients with Alzheimer's disease may be particularly susceptible to adverse effects of statins. Dement Geriatr Cogn Disord. 2004; 17(3): 109-16.

3. Allan CL, Behrman S, Ebmeier KP, Valkanova V. Diagnosing early cognitive decline-When, how and for whom? Maturitas. 2017; 96: 103-8.

4. Almuti K, Rimawi R, Spevack D, Ostfeld RJ. Effects of statins beyond lipid lowering: potential for clinical benefits. Int J Cardiol. 2006; 109(1): 7-15.

5. Austen B, Christodoulou G, Terry JE. Relation between cholesterol levels, statins and Alzheimer's disease in the human population. The journal of nutrition, health & aging. 2002; 6(6): 377-82.

6. Baghdasarian SB, Jneid H, Hoogwerf BJ. Association of dyslipidemia and effects of statins on nonmacrovascular diseases. Clin Ther. 2004; 26(3): 337-51.

7. Barone E, Di Domenico F, Butterfield DA. Statins more than cholesterol lowering agents in Alzheimer disease: their pleiotropic functions as potential therapeutic targets. Biochem Pharmacol. 2014; 88(4): 605-16.

8. Butterfield DA. Atorvastatin and Abeta(1-40): not as simple as cholesterol reduction in brain and relevance to Alzheimer disease. Exp Neurol. 2011; 228(1): 15-8.

9. Caballero J, Nahata M. Do statins slow down Alzheimer's disease? A review. Journal of clinical pharmacy and therapeutics. 2004; 29(3): 209-13.

10. Chatterjee S, Krishnamoorthy P, Ranjan P, Roy A, Chakraborty A, Sabharwal MS, et al. Statins and cognitive function: an updated review. Current cardiology reports. 2015; 17(2): 4.

11. Crisby M. The role of pleiotropic effects of statins in dementia. Acta Neurologica Scandinavica Supplementum. 2006; 114: 115-8.

12. Crisby M, Carlson LA, Winblad B. Statins in the prevention and treatment of Alzheimer disease. Alzheimer disease and associated disorders. 2002; 16(3): 131-6.

13. Daneschvar HL, Aronson MD, Smetana GW. Do statins prevent Alzheimer's disease? A narrative review. European journal of internal medicine. 2015; 26(9): 666-9.

14. Davignon J, Leiter LA. Ongoing clinical trials of the pleiotropic effects of statins. Vascular health and risk management. 2005; 1(1): 29-40.

15. Desai CS, Martin SS, Blumenthal RS. Non-cardiovascular effects associated with statins. Bmj. 2014; 349: g3743.

16. Giannopoulos S, Katsanos AH, Kosmidou M, Tsivgoulis G. Statins and vascular dementia: a review. J Alzheimers Dis. 2014; 42 Suppl 3: S315-20.

17. Gnjidic D, Naganathan V, Freedman SB, Beer CE, McLachlan AJ, Figtree GA, et al. Statin Therapy and Cognition in Older People: What is the Evidence? Current clinical pharmacology. 2015; 10(3): 185-93.

18. Gracias G, Garrison S, Allan GM. Statins and cognitive impairment. Canadian family physician Medecin de famille canadien. 2014; 60(8): e391.

19. Hamel E, Royea J, Ongali B, Tong XK. Neurovascular and Cognitive failure in Alzheimer's Disease: Benefits of Cardiovascular Therapy. Cellular and molecular neurobiology. 2016; 36(2): 219-32.

20. Hoglund K, Blennow K. Effect of HMG-CoA reductase inhibitors on beta-amyloid peptide levels: implications for Alzheimer's disease. CNS Drugs. 2007; 21(6): 449-62.

21. Hoglund K, Wallin A, Blennow K. Effect of statins on beta-amyloid metabolism in humans: potential importance for the development of senile plaques in Alzheimer's disease. Acta Neurol Scand Suppl. 2006; 185: 87-92.

22. Jedrziewski MK, Lee VM, Trojanowski JQ. Lowering the risk of Alzheimer's disease: evidence-based practices emerge from new research. Alzheimers Dement. 2005; 1(2): 152-60.

23. Kandiah N, Feldman HH. Therapeutic potential of statins in Alzheimer's disease. J Neurol Sci. 2009; 283(1-2): 230-4.

24. Larson EB. ACP Journal Club. Review: In older persons at vascular risk, statins do not prevent dementia or cognitive decline at 3.5 to 5 years. Ann Intern Med. 2016; 164(8): Jc42.

25. Ling Q, Tejada-Simon MV. Statins and the brain: New perspective for old drugs. Progress in neuro-psychopharmacology & biological psychiatry. 2016; 66: 80-6.

26. Livingstone N, Hanratty J, McShane R, Macdonald G. Pharmacological interventions for cognitive decline in people with Down syndrome. The Cochrane database of systematic reviews. 2015; (10): Cd011546.

27. Malfitano AM, Marasco G, Proto MC, Laezza C, Gazzerro P, Bifulco M. Statins in neurological disorders: an overview and update. Pharmacological research. 2014; 88: 74-83.

28. McGuinness B, Craig D, Bullock R, Malouf R, Passmore P. Statins for the treatment of dementia. The Cochrane database of systematic reviews. 2014; (7): Cd007514.

29. McGuinness B, Craig D, Bullock R, Passmore P. Statins for the prevention of dementia. The Cochrane database of systematic reviews. 2009; (2): Cd003160.

30. McGuinness B, Craig D, Bullock R, Passmore P. Statins for the prevention of dementia. The Cochrane database of systematic reviews. 2016; (1): Cd003160.

31. McGuinness B, O'Hare J, Craig D, Bullock R, Malouf R, Passmore P. Cochrane review on 'Statins for the treatment of dementia'. Int J Geriatr Psychiatry. 2013; 28(2): 119-26.

32. McGuinness B, Passmore P. Can statins prevent or help treat Alzheimer's disease? J Alzheimers Dis. 2010; 20(3): 925-33.

33. Mendoza-Oliva A, Zepeda A, Arias C. The complex actions of statins in brain and their relevance for Alzheimer's disease treatment: an analytical review. Curr Alzheimer Res. 2014; 11(9): 817-33.

34. Menezes AR, Lavie CJ, Milani RV, O'Keefe J. The effects of statins on prevention of stroke and dementia: a review. Journal of cardiopulmonary rehabilitation and prevention. 2012; 32(5): 240-9.

35. Merlo S, Spampinato S, Canonico PL, Copani A, Sortino MA. Alzheimer's disease: brain expression of a metabolic disorder? Trends in endocrinology and metabolism: TEM. 2010; 21(9): 537-44.

36. Miida T, Takahashi A, Ikeuchi T. Prevention of stroke and dementia by statin therapy: experimental and clinical evidence of their pleiotropic effects. Pharmacology & therapeutics. 2007; 113(2): 378-93.

37. Milani RV. Lipid and statin effects on stroke and dementia. The American journal of geriatric cardiology. 2004; 13(3 Suppl 1): 25-8.

38. Muangpaisan W, Brayne C, Alzheimer's Society Vascular Dementia Systematic Review G. Systematic review of statins for the prevention of vascular dementia or dementia. Geriatr Gerontol Int. 2010; 10(2): 199-208.

39. Pac-Soo C, Lloyd DG, Vizcaychipi MP, Ma D. Statins: the role in the treatment and prevention of Alzheimer's neurodegeneration. J Alzheimers Dis. 2011; 27(1): 1-10.

40. Power MC, Weuve J, Sharrett AR, Blacker D, Gottesman RF. Statins, cognition, and dementia-systematic review and methodological commentary. Nature reviews Neurology. 2015; 11(4): 220-9.

41. Reid PC, Urano Y, Kodama T, Hamakubo T. Alzheimer's disease: cholesterol, membrane rafts, isoprenoids and statins. Journal of cellular and molecular medicine. 2007; 11(3): 383-92.

42. Reiss AB, Wirkowski E. Statins in neurological disorders: mechanisms and therapeutic value. TheScientificWorldJournal. 2009; 9: 1242-59.

43. Reitz C. Dyslipidemia and dementia: current epidemiology, genetic evidence, and mechanisms behind the associations. J Alzheimers Dis. 2012; 30 Suppl 2: S127-45.

44. Rockwood K. Epidemiological and clinical trials evidence about a preventive role for statins in Alzheimer's disease. Acta Neurologica Scandinavica Supplementum. 2006; 114: 71-7.

45. Rockwood K, Darvesh S. The risk of dementia in relation to statins and other lipid lowering agents. Neurological research. 2003; 25(6): 601-4.

46. Rojas-Fernandez CH, Cameron JC. Is statin-associated cognitive impairment clinically relevant? A narrative review and clinical recommendations. The Annals of pharmacotherapy. 2012; 46(4): 549-57.

47. Samaras K, Brodaty H, Sachdev PS. Does statin use cause memory decline in the elderly? Trends in cardiovascular medicine. 2016; 26(6): 550-65.

48. Scott HD, Laake K. Statins for the prevention of Alzheimer's disease. The Cochrane database of systematic reviews. 2001; (4): Cd003160.

49. Shah NP, Swiger KJ, Martin SS. Impact on cognitive function-are all statins the same? Current atherosclerosis reports. 2015; 17(1): 466.

50. Silva T, Teixeira J, Remiao F, Borges F. Alzheimer's disease, cholesterol, and statins: the junctions of important metabolic pathways. Angewandte Chemie (International ed in English). 2013; 52(4): 1110-21.

51. Simons M, Keller P, Dichgans J, Schulz JB. Cholesterol and Alzheimer's disease: is there a link? Neurology. 2001; 57(6): 1089-93.

52. Smith DA. ACP Journal Club. Review: statins are not associated with cognitive impairment or dementia in cognitively intact adults. Ann Intern Med. 2014; 160(10): Jc11, jc0.

53. Sparks DL, Martin TA, Gross DR, Hunsaker JC, 3rd. Link between heart disease, cholesterol, and Alzheimer's disease: a review. Microscopy research and technique. 2000; 50(4): 287-90.

54. Sparks DL, Sabbagh M, Connor D, Soares H, Lopez J, Stankovic G, et al. Statin therapy in Alzheimer's disease. Acta Neurol Scand Suppl. 2006; 185: 78-86.

55. Stepien K, Tomaszewski M, Czuczwar SJ. Neuroprotective properties of statins. Pharmacological reports : PR. 2005; 57(5): 561-9.

56. Waldman A, Kritharides L. The pleiotropic effects of HMG-CoA reductase inhibitors: their role in osteoporosis and dementia. Drugs. 2003; 63(2): 139-52.

57. Wanamaker BL, Swiger KJ, Blumenthal RS, Martin SS. Cholesterol, statins, and dementia: what the cardiologist should know. Clinical cardiology. 2015; 38(4): 243-50.

58. Whitfield JF. Can statins put the brakes on Alzheimer's disease? Expert opinion on investigational drugs. 2006; 15(12): 1479-85.

59. Wolozin B. Cholesterol, statins and dementia. Current opinion in lipidology. 2004; 15(6): 667-72.

60. Wolozin B. Cholesterol and the biology of Alzheimer's disease. Neuron. 2004; 41(1): 7-10.

61. Wolozin B, Brown J, 3rd, Theisler C, Silberman S. The cellular biochemistry of cholesterol and statins: insights into the pathophysiology and therapy of Alzheimer's disease. CNS drug reviews. 2004; 10(2): 127-46.

62. Xiong GL, Benson A, Doraiswamy PM. Statins and cognition: what can we learn from existing randomized trials? CNS spectrums. 2005; 10(11): 867-74.

63. Zipp F, Waiczies S, Aktas O, Neuhaus O, Hemmer B, Schraven B, et al. Impact of HMG-CoA reductase inhibition on brain pathology. Trends in pharmacological sciences. 2007; 28(7): 342-9.

64. Bunt CW, Hogan AJ. The Effect of Statins on Dementia and Cognitive Decline. American family physician. 2017; 95(3): 151-2.

65. Santos CY, Snyder PJ, Wu WC, Zhang M, Echeverria A, Alber J. Pathophysiologic relationship between Alzheimer's disease, cerebrovascular disease, and cardiovascular risk: A review and synthesis. Alzheimers Dement (Amst). 2017; 7: 69-87.

66. Hersi M, Irvine B, Gupta P, Gomes J, Birkett N, Krewski D. Risk factors associated with the onset and progression of Alzheimer's disease: A systematic review of the evidence. Neurotoxicology. 2017; 61: 143-87.

67. Stephenson NE, Crowe SF. Statin Use, Ageing, and Cognition: A Review. Australian Psychologist. 2016; 51(3): 188-205.

68. Kelley B, Glasser S. Cognitive Effects of Statin Medications. CNS Drugs. 2014; 28(5): 411-9.

69. Antuono P. Response to The UCSD Statin Study: a randomized controlled trial assessing the impact of statins on selected noncardiac outcomes". Contemporary clinical trials. 2005; 26(3): 417-8.

70. Arvanitakis Z, Knopman DS. Clinical trial efforts in Alzheimer disease: why test statins? Neurology. 2010; 74(12): 945-6.

71. Birkenhager WH, Wang JG, Staessen JA. Dementia and statins. Lancet. 2001; 357(9259): 880; author reply -1.

72. Bollen EL, Gaw A, Buckley BM. Statin therapy and the prevention of dementia. Arch Neurol. 2001; 58(6): 1023-4.

73. Goldstein MR, Mascitelli L, Pezzetta F, Haan MN, Cramer C, Kalbfleisch J, et al. Use of statins and incidence of dementia and cognitive impairment without dementia in a cohort study. Neurology. 2009; 72(13): 1190-1.

74. Haan MN. Can statins prevent dementia in older adults? J Am Geriatr Soc. 2010; 58(7): 1393-4.

75. Kawada T. Statins use and dementia: causality of the association. Journal of internal medicine. 2015; 277(5): 624.

76. Lavie CJ, DiNicolantonio JJ, O'Keefe JH, Milani RV. Do statins cause or prevent dementia? Eur J Neurol. 2015; 22(6): 885-6.

77. Lavie CJ, Milani RV. Optimal lipids, statins, and dementia. J Am Coll Cardiol. 2005; 45(6): 963-4; author reply 4-5.

78. Lesser GT, Libow LS. Statin-Alzheimer disease association not yet proven. Arch Neurol. 2001; 58(6): 1022-3.

79. Lowe G, Rumley A, Packard C, Shepherd J. Dementia and statins. Lancet. 2001; 357(9259): 881.

80. Muldoon MF. Report on statins and dementia disputed. Arch Neurol. 2001; 58(7): 1166-7.

81. Beyreuther K HTSSittoAsdEN, Supplement 4:S116.

82. Breitner JCS ZP, Li G, Rea TD, Psaty BM. S1-03-01 Can statins prevent AD, or are they just prescribed less often to those with cognitive disorders? Neurobiology of Aging. 2004; 25, supplement 2: S5.

83. Silbert LC. Does statin use decrease the amount of Alzheimer disease pathology in the brain? Neurology. 2007; 69(9): E8-11.

84. Sparks DL. Alzheimer disease: statins in the treatment of Alzheimer disease. Nature reviews Neurology. 2011; 7(12): 662-3.

85. Sparks L. Statins and cognitive function. Journal of neurology, neurosurgery, and psychiatry. 2009; 80(1): 1-2.

86. Vos E, Nehrlich HH. Use of statins and incidence of dementia and cognitive impairment without dementia in a cohort study. Neurology. 2009; 73(5): 406; author reply -7.

87. Winblad B CL, Crisby M. Statins in the treatment of Alzheimers Disease. . European Neuropsychopharmacology 2001; 11: S328-S9.

88. Etminan M, Gill S, Samii A. The role of lipid-lowering drugs in cognitive function: a meta-analysis of observational studies. Pharmacotherapy. 2003; 23(6): 726-30.

89. Liang T, Li R, Cheng O. Statins for Treating Alzheimer's Disease: Truly Ineffective? Eur Neurol. 2015; 73(5-6): 360-6.

90. Macedo AF, Taylor FC, Casas JP, Adler A, Prieto-Merino D, Ebrahim S. Unintended effects of statins from observational studies in the general population: systematic review and meta-analysis. BMC Med. 2014; 12: 51.

91. Ott BR, Daiello LA, Dahabreh IJ, Springate BA, Bixby K, Murali M, et al. Do statins impair cognition? A systematic review and meta-analysis of randomized controlled trials. Journal of general internal medicine. 2015; 30(3): 348-58.

92. Pandey RD, Gupta PP, Jha D, Kumar S. Role of statins in Alzheimer's disease: a retrospective meta-analysis for commonly investigated clinical parameters in RCTs. International Journal of Neuroscience. 2013; 123(8): 521-5.

93. Richardson K, Schoen M, French B, Umscheid CA, Mitchell MD, Arnold SE, et al. Statins and cognitive function: a systematic review. Ann Intern Med. 2013; 159(10): 688-97.

94. Song Y, Nie H, Xu Y, Zhang L, Wu Y. Association of statin use with risk of dementia: a meta-analysis of prospective cohort studies. Geriatr Gerontol Int. 2013; 13(4): 817-24.

95. Swiger KJ, Manalac RJ, Blumenthal RS, Blaha MJ, Martin SS. Statins and cognition: a systematic review and meta-analysis of short- and long-term cognitive effects. Mayo Clin Proc. 2013; 88(11): 1213-21.

96. Wong WB, Lin VW, Boudreau D, Devine EB. Statins in the prevention of dementia and Alzheimer's disease: a meta-analysis of observational studies and an assessment of confounding. Pharmacoepidemiol Drug Saf. 2013; 22(4): 345-58.

97. Zhou B, Teramukai S, Fukushima M. Prevention and treatment of dementia or Alzheimer's disease by statins: a meta-analysis. Dement Geriatr Cogn Disord. 2007; 23(3): 194-201.

98. Bellou V, Belbasis L, Tzoulaki I, Middleton LT, Ioannidis JP, Evangelou E. Systematic evaluation of the associations between environmental risk factors and dementia: An umbrella review of systematic reviews and meta-analyses. Alzheimers Dement. 2017; 13(4): 406-18.

99. Trompet S, van Vliet P, de Craen AJ, Jolles J, Buckley BM, Murphy MB, et al. Pravastatin and cognitive function in the elderly. Results of the PROSPER study. J Neurol. 2010; 257(1): 85-90.

100. Hosomi N, Nagai Y, Kohriyama T, Ohtsuki T, Aoki S, Nezu T, et al. The Japan Statin Treatment Against Recurrent Stroke (J-STARS): A Multicenter, Randomized, Open-label, Parallel-group Study. EBioMedicine. 2015; 2(9): 1071-8.

101. Shepherd J, Blauw GJ, Murphy MB, Bollen EL, Buckley BM, Cobbe SM, et al. Pravastatin in elderly individuals at risk of vascular disease (PROSPER): a randomised controlled trial. Lancet. 2002; 360(9346): 1623-30.

102. Heart Protection Study Collaborative G. MRC/BHF Heart Protection Study of cholesterol lowering with simvastatin in 20,536 high-risk individuals: a randomised placebo-controlled trial. Lancet. 2002; 360(9326): 7-22.

103. Ridker PM, Cannon CP, Morrow D, Rifai N, Rose LM, McCabe CH, et al. C-reactive protein levels and outcomes after statin therapy. N Engl J Med. 2005; 352(1): 20-8.

104. Collins R, Armitage J, Parish S, Sleight P, Peto R, Heart Protection Study Collaborative G. Effects of cholesterol-lowering with simvastatin on stroke and other major vascular events in 20536 people with cerebrovascular disease or other high-risk conditions. Lancet. 2004; 363(9411): 757-67.

105. Benito-Leon J, Louis ED, Vega S, Bermejo-Pareja F. Statins and cognitive functioning in the elderly: a population-based study. J Alzheimers Dis. 2010; 21(1): 95-102.

106. Chao TF, Liu CJ, Chen SJ, Wang KL, Lin YJ, Chang SL, et al. Statins and the risk of dementia in patients with atrial fibrillation: A nationwide population-based cohort study. Int J Cardiol. 2015; 196: 91-7.

107. Chen PY, Liu SK, Chen CL, Wu CS. Long-term statin use and dementia risk in Taiwan. Journal of geriatric psychiatry and neurology. 2014; 27(3): 165-71.

108. Chou CY, Chou YC, Chou YJ, Yang YF, Huang N. Statin use and incident dementia: a nationwide cohort study of Taiwan. Int J Cardiol. 2014; 173(2): 305-10.

109. Chuang CS, Lin CL, Lin MC, Sung FC, Kao CH. Decreased prevalence of dementia associated with statins: a national population-based study. European Journal of Neurology. 2015; 22(6): 912-8.

110. Fassbender K, Stroick M, Bertsch T, Ragoschke A, Kuehl S, Walter S, et al. Effects of statins on human cerebral cholesterol metabolism and secretion of Alzheimer amyloid peptide. Neurology. 2002; 59(8): 1257-8.

111. Green RC, McNagny SE, Jayakumar P, Cupples LA, Benke K, Farrer LA, et al. Statin use and the risk of Alzheimer's disease: the MIRAGE study. Alzheimers Dement. 2006; 2(2): 96-103.

112. Hajjar I, Schumpert J, Hirth V, Wieland D, Eleazer GP. The impact of the use of statins on the prevalence of dementia and the progression of cognitive impairment. J Gerontol A Biol Sci Med Sci. 2002; 57(7): M414-8.

113. Heymann AD, Ravona-Springer R, Moshier EL, Godbold J, Beeri MS. Statin Use is Associated with Better Cognitive Function in Elderly with Type 2 Diabetes. J Alzheimers Dis. 2015; 47(1): 55-9.

114. Jick H, Zornberg GL, Jick SS, Seshadri S, Drachman DA. Statins and the risk of dementia. Lancet. 2000; 356(9242): 1627-31.

115. Lin FC, Chuang YS, Hsieh HM, Lee TC, Chiu KF, Liu CK, et al. Early Statin Use and the Progression of Alzheimer Disease: A Total Population-Based Case-Control Study. Medicine. 2015; 94(47): e2143.

116. Riordan HJ SV, Zdon GS, Anderson DR, Seeger J, Schaeffer J, et al. P4-100: Statin use and the risk of Alzheimer's disease and amyotrophic lateral sclerosis: Insights from a health insurance database. . Alzheimer's & Dementia. 2008; 4: T698.

117. K. R. Use of lipid lowering agents and the risk of cognitive impairment not meeting dementia criteria in relation to apolipoprotein E status. . Alzheimer's & Dementia 2005; 1: S66.

118. Rockwood K, Howlett S, Fisk J, Darvesh S, Tuokko H, Hogan DB, et al. Lipid-lowering agents and the risk of cognitive impairment that does not meet criteria for dementia, in relation to apolipoprotein E status. Neuroepidemiology. 2007; 29(3-4): 201-7.

119. Rockwood K, Kirkland S, Hogan DB, MacKnight C, Merry H, Verreault R, et al. Use of lipid-lowering agents, indication bias, and the risk of dementia in community-dwelling elderly people. Arch Neurol. 2002; 59(2): 223-7.

120. Wu CK, Yang YH, Lin TT, Tsai CT, Hwang JJ, Lin JL, et al. Statin use reduces the risk of dementia in elderly patients: a nationwide data survey and propensity analysis. Journal of internal medicine. 2015; 277(3): 343-52.

121. Yang YH, Teng HW, Lai YT, Li SY, Lin CC, Yang AC, et al. Statins Reduces the Risk of Dementia in Patients with Late-Onset Depression: A Retrospective Cohort Study. PloS one. 2015; 10(9): e0137914.

122. Zamrini E, McGwin G, Roseman JM. Association between statin use and Alzheimer's disease. Neuroepidemiology. 2004; 23(1-2): 94-8.

123. Parale GP, Baheti NN, Kulkarni PM, Panchal NV. Effects of atorvastatin on higher functions. Eur J Clin Pharmacol. 2006; 62(4): 259-65.

124. Blasko I, Kemmler G, Krampla W, Jungwirth S, Wichart I, Jellinger K, et al. Plasma amyloid beta protein 42 in non-demented persons aged 75 years: effects of concomitant medication and medial temporal lobe atrophy. Neurobiol Aging. 2005; 26(8): 1135-43.

125. Carlsson CM, Nondahl DM, Klein BE, McBride PE, Sager MA, Schubert CR, et al. Increased atherogenic lipoproteins are associated with cognitive impairment: effects of statins and subclinical atherosclerosis. Alzheimer disease and associated disorders. 2009; 23(1): 11-7.

126. Doraiswamy PM, Steffens DC, McQuoid DR. Statin use and hippocampal volumes in elderly subjects at risk for Alzheimer's disease: a pilot observational study. American journal of Alzheimer's disease and other dementias. 2004; 19(5): 275-8.

127. Dufouil C, Richard F, Fievet N, Dartigues JF, Ritchie K, Tzourio C, et al. APOE genotype, cholesterol level, lipid-lowering treatment, and dementia: the Three-City Study. Neurology. 2005; 64(9): 1531-8.

128. Rodriguez EG, Dodge HH, Birzescu MA, Stoehr GP, Ganguli M. Use of lipid-lowering drugs in older adults with and without dementia: a community-based epidemiological study. J Am Geriatr Soc. 2002; 50(11): 1852-6.

129. Wolozin B, Kellman W, Ruosseau P, Celesia GG, Siegel G. Decreased prevalence of Alzheimer disease associated with 3-hydroxy-3-methyglutaryl coenzyme A reductase inhibitors. Arch Neurol. 2000; 57(10): 1439-43.

130. . !!! INVALID CITATION !!!

131. Sparks DL, Kryscio RJ, Connor DJ, Sabbagh MN, Sparks LM, Lin Y, et al. Cholesterol and cognitive performance in normal controls and the influence of elective statin use after conversion to mild cognitive impairment: results in a clinical trial cohort. Neuro-degenerative diseases. 2010; 7(1-3): 183-6.

132. Sparks DL, Lopez J, Connor D, Sabbagh M, Seward J, Browne P. A position paper: based on observational data indicating an increased rate of altered blood chemistry requiring withdrawal from the Alzheimer's Disease Cholesterol-Lowering Treatment Trial (ADCLT). Journal of molecular neuroscience : MN. 2003; 20(3): 407-10.

133. Sparks DL, Sabbagh MN, Breitner JC, Hunsaker JC, 3rd. Is cholesterol a culprit in Alzheimer's disease? Int Psychogeriatr. 2003; 15 Suppl 1: 153-9.

134. Sparks DL, Sabbagh MN, Connor DJ, Lopez J, Launer LJ, Browne P, et al. Atorvastatin for the treatment of mild to moderate Alzheimer disease: preliminary results. Arch Neurol. 2005; 62(5): 753-7.

135. Stankovic G, Sparks DL. Change in circulating C-reactive protein is not associated with atorvastatin treatment in Alzheimer's disease. Neurological research. 2006; 28(6): 621-4.

136. Varol E. Antihypertensive drugs and statins must be considered in arterial stiffness evaluation in patients with Alzheimer's disease. Neurological Sciences. 2016; 37(8): 1367-.

137. Winblad B, Jelic V, Kershaw P, Amatniek J. Effects of statins on cognitive function in patients with Alzheimer's disease in galantamine clinical trials. Drugs Aging. 2007; 24(1): 57-61.

138. Geifman N, Brinton RD, Kennedy RE, Schneider LS, Butte AJ. Evidence for benefit of statins to modify cognitive decline and risk in Alzheimer's disease. Alzheimers Res Ther. 2017; 9(1): 10.

139. Smith KB, Kang P, Sabbagh MN, Alzheimer's Disease Neuroimaging I. The Effect of Statins on Rate of Cognitive Decline in Mild Cognitive Impairment. Alzheimers Dement (N Y). 2017; 3(2): 149-56.

140. Ellul J, Archer N, Foy CM, Poppe M, Boothby H, Nicholas H, et al. The effects of commonly prescribed drugs in patients with Alzheimer's disease on the rate of deterioration. Journal of neurology, neurosurgery, and psychiatry. 2007; 78(3): 233-9.

141. Feldman HH, Doody RS, Kivipelto M, Sparks DL, Waters DD, Jones RW, et al. Randomized controlled trial of atorvastatin in mild to moderate Alzheimer disease: LEADe. Neurology. 2010; 74(12): 956-64.

142. Jones RW, Kivipelto M, Feldman H, Sparks L, Doody R, Waters DD, et al. The Atorvastatin/Donepezil in Alzheimer's Disease Study (LEADe): design and baseline characteristics. Alzheimers Dement. 2008; 4(2): 145-53.

143. Lilly SM, Mortensen EM, Frei CR, Pugh MJ, Mansi IA. Comparison of the risk of psychological and cognitive disorders between persistent and nonpersistent statin users. Am J Cardiol. 2014; 114(7): 1035-9.

144. Liu JC, Hsu YP, Kao PF, Hao WR, Liu SH, Lin CF, et al. Influenza Vaccination Reduces Dementia Risk in Chronic Kidney Disease Patients: A Population-Based Cohort Study. Medicine. 2016; 95(9): e2868.

145. Masse I, Bordet R, Deplanque D, Al Khedr A, Richard F, Libersa C, et al. Lipid lowering agents are associated with a slower cognitive decline in Alzheimer's disease. Journal of neurology, neurosurgery, and psychiatry. 2005; 76(12): 1624-9.

146. Padala KP KB, Potter JF. A Nursing Home Study of Impact of Statins on Cognition. Journal of the American Medical Directors Association 2008; 9: B3.

147. Padala KP, Padala PR, McNeilly DP, Geske JA, Sullivan DH, Potter JF. The effect of HMG-CoA reductase inhibitors on cognition in patients with Alzheimer's dementia: a prospective withdrawal and rechallenge pilot study. The American journal of geriatric pharmacotherapy. 2012; 10(5): 296-302.

148. Pressman P, Gottfried JA. Journal Club: a randomized, double-blind, placebo-controlled trial of simvastatin to treat Alzheimer disease. Neurology. 2012; 79(4): e33-6.

149. M. S. S5-01-05: Multi-center, randomized, double-blind, placebo-controlled trial of Simvastatin to slow the progression of Alzheimer's disease. . Alzheimer's & Dementia. 2008; 4: T200.

150. Sano M, Bell KL, Galasko D, Galvin JE, Thomas RG, van Dyck CH, et al. A randomized, double-blind, placebo-controlled trial of simvastatin to treat Alzheimer disease. Neurology. 2011; 77(6): 556-63.

151. Smeeth L, Douglas I, Hall AJ, Hubbard R, Evans S. Effect of statins on a wide range of health outcomes: a cohort study validated by comparison with randomized trials. Br J Clin Pharmacol. 2009; 67(1): 99-109.

152. Simons M, Schwarzler F, Lutjohann D, von Bergmann K, Beyreuther K, Dichgans J, et al. Treatment with simvastatin in normocholesterolemic patients with Alzheimer's disease: A 26-week randomized, placebo-controlled, double-blind trial. Ann Neurol. 2002; 52(3): 346-50.

153. Sjogren M, Gustafsson K, Syversen S, Olsson A, Edman A, Davidsson P, et al. Treatment with simvastatin in patients with Alzheimer's disease lowers both alpha- and beta-cleaved amyloid precursor protein. Dement Geriatr Cogn Disord. 2003; 16(1): 25-30.

154. Soares HD RJ, Sparks L. . P-100: Identification of biomarkers responsive to statin treatment in Alzheimer’s disease. Alzheimer's & Dementia. 2007; 3: S130.

155. Sparks DL, Connor DJ, Sabbagh MN, Petersen RB, Lopez J, Browne P. Circulating cholesterol levels, apolipoprotein E genotype and dementia severity influence the benefit of atorvastatin treatment in Alzheimer's disease: results of the Alzheimer's Disease Cholesterol-Lowering Treatment (ADCLT) trial. Acta Neurologica Scandinavica Supplementum. 2006; 114: 3-7.

156. Blasko I, Jungwirth S, Jellinger K, Kemmler G, Krampla W, Weissgram S, et al. Effects of medications on plasma amyloid beta (Abeta) 42: longitudinal data from the VITA cohort. Journal of psychiatric research. 2008; 42(11): 946-55.

157. Buxbaum JD, Cullen EI, Friedhoff LT. Pharmacological concentrations of the HMG-CoA reductase inhibitor lovastatin decrease the formation of the Alzheimer beta-amyloid peptide in vitro and in patients. Frontiers in bioscience : a journal and virtual library. 2002; 7: a50-9.

158. Carlsson CM, Xu G, Wen Z, Barnet JH, Blazel HM, Chappell RJ, et al. Effects of atorvastatin on cerebral blood flow in middle-aged adults at risk for Alzheimer's disease: a pilot study. Curr Alzheimer Res. 2012; 9(8): 990-7.

159. Chodick G, Shalev V, Gerber Y, Heymann AD, Silber H, Simah V, et al. Long-term persistence with statin treatment in a not-for-profit health maintenance organization: a population-based retrospective cohort study in Israel. Clin Ther. 2008; 30(11): 2167-79.

160. Evans BA, Evans JE, Baker SP, Kane K, Swearer J, Hinerfeld D, et al. Long-term statin therapy and CSF cholesterol levels: implications for Alzheimer's disease. Dement Geriatr Cogn Disord. 2009; 27(6): 519-24.

161. Evans MA, Golomb BA. Statin-associated adverse cognitive effects: survey results from 171 patients. Pharmacotherapy. 2009; 29(7): 800-11.

162. Friedhoff LT, Cullen EI, Geoghagen NS, Buxbaum JD. Treatment with controlled-release lovastatin decreases serum concentrations of human beta-amyloid (A beta) peptide. Int J Neuropsychopharmacol. 2001; 4(2): 127-30.

163. Giri J, McDermott MM, Greenland P, Guralnik JM, Criqui MH, Liu K, et al. Statin use and functional decline in patients with and without peripheral arterial disease. J Am Coll Cardiol. 2006; 47(5): 998-1004.

164. Glodzik L BV. The effects of statin use on Alzheimer's disease CSF biomarkers in cognitively healthy elderly. . Alzheimer's & Dementia. 2011; 7: e58.

165. Hoglund K, Syversen S, Lewczuk P, Wallin A, Wiltfang J, Blennow K. Statin treatment and a disease-specific pattern of beta-amyloid peptides in Alzheimer's disease. Experimental brain research. 2005; 164(2): 205-14.

166. Hoglund K, Thelen KM, Syversen S, Sjogren M, von Bergmann K, Wallin A, et al. The effect of simvastatin treatment on the amyloid precursor protein and brain cholesterol metabolism in patients with Alzheimer's disease. Dement Geriatr Cogn Disord. 2005; 19(5-6): 256-65.

167. Horsdal HT, Olesen AV, Gasse C, Sorensen HT, Green RC, Johnsen SP. Use of statins and risk of hospitalization with dementia: a Danish population-based case-control study. Alzheimer disease and associated disorders. 2009; 23(1): 18-22.

168. Ishii K, Tokuda T, Matsushima T, Miya F, Shoji S, Ikeda S, et al. Pravastatin at 10 mg/day does not decrease plasma levels of either amyloid-beta (Abeta) 40 or Abeta 42 in humans. Neurosci Lett. 2003; 350(3): 161-4.

169. Riekse RG, Li G, Petrie EC, Leverenz JB, Vavrek D, Vuletic S, et al. Effect of statins on Alzheimer's disease biomarkers in cerebrospinal fluid. J Alzheimers Dis. 2006; 10(4): 399-406.

170. Suribhatla S, Dennis MS, Potter JF. A study of statin use in the prevention of cognitive impairment of vascular origin in the UK. J Neurol Sci. 2005; 229-230: 147-50.

171. Young-Xu Y, Chan KA, Liao JK, Ravid S, Blatt CM. Long-term statin use and psychological well-being. J Am Coll Cardiol. 2003; 42(4): 690-7.

172. Tokuda T, Tamaoka A, Matsuno S, Sakurai S, Shimada H, Morita H, et al. Plasma levels of amyloid beta proteins did not differ between subjects taking statins and those not taking statins. Ann Neurol. 2001; 49(4): 546-7.

173. Li G, Mayer CL, Morelli D, Millard SP, Raskind WH, Petrie EC, et al. Effect of simvastatin on CSF Alzheimer disease biomarkers in cognitively normal adults. Neurology. 2017; 89(12): 1251-5.

174. Ou HT, Chang KC, Li CY, Yang CY, Ko NY. Intensive statin regimens for reducing risk of cardiovascular diseases among human immunodeficiency virus-infected population: A nation-wide longitudinal cohort study 2000-2011. Int J Cardiol. 2017; 230: 592-8.

175. Chung CM, Lin MS, Chang CH, Cheng HW, Chang ST, Wang PC, et al. Moderate to high intensity statin in dialysis patients after acute myocardial infarction: A national cohort study in Asia. Atherosclerosis. 2017; 267: 158-66.

176. Proietti M, Laroche C, Nyvad O, Haberka M, Vassilikos VP, Maggioni AP, et al. Use of statins and adverse outcomes in patients with atrial fibrillation: An analysis from the EURObservational Research Programme Atrial Fibrillation (EORP-AF) general registry pilot phase. Int J Cardiol. 2017; 248: 166-72.

177. Lee MG, Lee CC, Lai CC, Hsu TC, Porta L, Lee M, et al. Preadmission statin use improves the outcome of less severe sepsis patients - a population-based propensity score matched cohort study. British journal of anaesthesia. 2017; 119(4): 645-54.

178. Proitsi P, Kim M, Whiley L, Simmons A, Sattlecker M, Velayudhan L, et al. Association of blood lipids with Alzheimer's disease: A comprehensive lipidomics analysis. Alzheimers Dement. 2017; 13(2): 140-51.

179. Robert H. Pietrzak SML, Yen Ying Lim, Sophie J. Bender, for the Australian Imaging, Biomarkers and Lifestyle Research Group. Plasma Cortisol, Brain Amyloid-β, and Cognitive Decline in Preclinical Alzheimer's Disease: A 6-Year Prospective Cohort Study. Biological Psychiatry: Cognitive Neuroscience and Neuroimaging,

. 2017; 2: 45-52.

180. Tjia J, Cutrona SL, Peterson D, Reed G, Andrade SE, Mitchell SL. Statin Discontinuation in Nursing Home Residents with Advanced Dementia. Journal of the American Geriatrics Society. 2014; 62(11): 2095-101.

181. Bernick C, Katz R, Smith NL, Rapp S, Bhadelia R, Carlson M, et al. Statins and cognitive function in the elderly: the Cardiovascular Health Study. Neurology. 2005; 65(9): 1388-94.

182. Hendrie H SJ, Gao S, Unverzagt FW, Murrell J, Hall KS. P4-385: Statin use protects against cognitive decline in African-American elderly. . Alzheimer's & Dementia 2006; 2: s631.

183. Steenland K, Zhao L, Goldstein FC, Levey AI. Statins and cognitive decline in older adults with normal cognition or mild cognitive impairment. J Am Geriatr Soc. 2013; 61(9): 1449-55.

184. Starr JM, McGurn B, Whiteman M, Pattie A, Whalley LJ, Deary IJ. Life long changes in cognitive ability are associated with prescribed medications in old age. International Journal of Geriatric Psychiatry. 2004; 19(4): 327-32.

185. Carlsson CM, Gleason CE, Hess TM, Moreland KA, Blazel HM, Koscik RL, et al. Effects of simvastatin on cerebrospinal fluid biomarkers and cognition in middle-aged adults at risk for Alzheimer's disease. J Alzheimers Dis. 2008; 13(2): 187-97.

186. Muldoon MF, Ryan CM, Sereika SM, Flory JD, Manuck SB. Randomized trial of the effects of simvastatin on cognitive functioning in hypercholesterolemic adults. The American journal of medicine. 2004; 117(11): 823-9.

187. Carlsson CM GC, Johnson SC, Xu G, Huang Y, Barnet J, et al. . A randomized, double-blind placebo-controlled trial of simvastatin on CSF, MRI, and cognitive biomarkers in middle-aged adults at risk for Alzheimer's disease: The ESPRIT Study. . Alzheimer's & Dementia. 2010; 6: S151-S2.

188. Posvar EL, Radulovic LL, Cilla DD, Jr., Whitfield LR, Sedman AJ. Tolerance and pharmacokinetics of single-dose atorvastatin, a potent inhibitor of HMG-CoA reductase, in healthy subjects. Journal of clinical pharmacology. 1996; 36(8): 728-31.

189. Muldoon MF, Barger SD, Ryan CM, Flory JD, Lehoczky JP, Matthews KA, et al. Effects of lovastatin on cognitive function and psychological well-being. The American journal of medicine. 2000; 108(7): 538-46.

190. Roth T, Richardson GR, Sullivan JP, Lee RM, Merlotti L, Roehrs T. Comparative effects of pravastatin and lovastatin on nighttime sleep and daytime performance. Clinical cardiology. 1992; 15(6): 426-32.

191. Santanello NC, Barber BL, Applegate WB, Elam J, Curtis C, Hunninghake DB, et al. Effect of pharmacologic lipid lowering on health-related quality of life in older persons: results from the Cholesterol Reduction in Seniors Program (CRISP) Pilot Study. J Am Geriatr Soc. 1997; 45(1): 8-14.

192. Gibellato MG, Moore JL, Selby K, Bower EA. Effects of lovastatin and pravastatin on cognitive function in military aircrew. Aviation, space, and environmental medicine. 2001; 72(9): 805-12.

193. Summers MJ, Oliver KR, Coombes JS, Fassett RG. Effect of atorvastatin on cognitive function in patients from the Lipid Lowering and Onset of Renal Disease (LORD) trial. Pharmacotherapy. 2007; 27(2): 183-90.

194. Carlsson CM, Papcke-Benson K, Carnes M, McBride PE, Stein JH. Health-related quality of life and long-term therapy with pravastatin and tocopherol (vitamin E) in older adults. Drugs Aging. 2002; 19(10): 793-805.

195. Cutler N, Sramek J, Veroff A, Block G, Stauffer L, Lines C. Effects of treatment with simvastatin and pravastatin on cognitive function in patients with hypercholesterolaemia. Br J Clin Pharmacol. 1995; 39(3): 333-6.

196. Gengo F, Cwudzinski D, Kinkel P, Block G, Stauffer L, Lines C. Effects of treatment with lovastatin and pravastatin on daytime cognitive performance. Clinical cardiology. 1995; 18(4): 209-14.

197. Harrison RW, Ashton CH. Do cholesterol-lowering agents affect brain activity? A comparison of simvastatin, pravastatin, and placebo in healthy volunteers. Br J Clin Pharmacol. 1994; 37(3): 231-6.

198. Kostis JB, Rosen RC, Wilson AC. Central nervous system effects of HMG CoA reductase inhibitors: lovastatin and pravastatin on sleep and cognitive performance in patients with hypercholesterolemia. Journal of clinical pharmacology. 1994; 34(10): 989-96.

199. Tendolkar I, Enajat M, Zwiers MP, van Wingen G, de Leeuw FE, van Kuilenburg J, et al. One-year cholesterol lowering treatment reduces medial temporal lobe atrophy and memory decline in stroke-free elderly with atrial fibrillation: evidence from a parallel group randomized trial. Int J Geriatr Psychiatry. 2012; 27(1): 49-58.

200. Berk-Planken I, de Konig I, Stolk R, Jansen H, Hoogerbrugge N. Atorvastatin, diabetic dyslipidemia, and cognitive functioning. Diabetes Care. 2002; 25(7): 1250-1.

201. Golomb BA DJ WH, Criqui MH. Do low dose statins affect cognition? Results of the UCSD statin study. . Circulation. 2006; 114: II289.

202. Jukema JW, Cannon CP, de Craen AJ, Westendorp RG, Trompet S. The controversies of statin therapy: weighing the evidence. J Am Coll Cardiol. 2012; 60(10): 875-81.

203. Hake AM GS, Lane K, Unverzagt F, Smith-Gamble V, Murrell J, et al. Statin use and incident dementia and Alzheimer's disease in elderly African-Americans. . Alzheimer's & Dementia. 2011; 7: S511-S2.

204. Zigman WB SN, Jenkins EC, Urv TK, Tycko B, Silverman W. . O2-04-01: Risk for Alzheimer’s disease in down syndrome is related to cholesterol level and statin use. . Alzheimer's & Dementia 2007; 3: S192.

205. Li L CD, Wan H, Chen J, Fukuchi K-i, Kim H, et al. O4-05-01 Enhancement of learning and memory by statins beyond Alzheimer's disease. Neurobiology of Aging. 2004; 25: S82.

206. Douma T, Borre Y, Hendriksen H, Olivier B, Oosting R. Simvastatin improves learning and memory in control but not in olfactory bulbectomized rats. Psychopharmacology. 2011; 216(4): 537-44.

207. Ble A, Hughes PM, Delgado J, Masoli JA, Bowman K, Zirk-Sadowski J, et al. Safety and Effectiveness of Statins for Prevention of Recurrent Myocardial Infarction in 12 156 Typical Older Patients: A Quasi-Experimental Study. J Gerontol A Biol Sci Med Sci. 2017; 72(2): 243-50.

208. Cooper SA, Caslake M, Evans J, Hassiotis A, Jahoda A, McConnachie A, et al. Toward onset prevention of cognitive decline in adults with Down syndrome (the TOP-COG study): study protocol for a randomized controlled trial. Trials. 2014; 15: 202.

209. Giugliano RP, Mach F, Zavitz K, Kurtz C, Schneider J, Wang H, et al. Design and rationale of the EBBINGHAUS trial: A phase 3, double-blind, placebo-controlled, multicenter study to assess the effect of evolocumab on cognitive function in patients with clinically evident cardiovascular disease and receiving statin background lipid-lowering therapy-A cognitive study of patients enrolled in the FOURIER trial. Clinical cardiology. 2017; 40(2): 59-65.

210. Gnjidic D, Fastbom J, Fratiglioni L, Rizzuto D, Angleman S, Johnell K. Statin Therapy and Dementia in Older Adults: Role of Disease Severity and Multimorbidity. J Am Geriatr Soc. 2016; 64(1): 223-4.

211. Wichmann MA, Cruickshanks KJ, Carlsson CM, Chappell R, Fischer ME, Klein BEK, et al. Long-Term Systemic Inflammation and Cognitive Impairment in a Population-Based Cohort. Journal of the American Geriatrics Society. 2014; 62(9): 1683-91.

212. Zissimopoulos JM, Barthold D, Brinton RD, Joyce G. Sex and Race Differences in the Association Between Statin Use and the Incidence of Alzheimer Disease. JAMA neurology. 2017; 74(2): 225-32.

213. Sinyavskaya L, Gauthier S, Renoux C, Dell'Aniello S, Suissa S, Brassard P. Comparative effect of statins on the risk of incident Alzheimer disease. Neurology. 2017.

Supplemental Table S3: Covariates considered in multivariable models in each included study.

Author (year)	Covariates considered in multivariable analyses	Numbers of adjust
Harding, 2017[35*]	Age, education, BMI, and PROCAM 10-year car diovascular risk	4
Chitnis, 2015[39]	Age, sex, number of all drugs, number of com orbid conditions, centers for medicare and Me dicaid services risk score, number of years in t he cohort, hospitalization, creatinine, LDL-C, an d HbAlc	10
Hendrie, 2015[40]	Age, sex, education, apoE, HTN, DM, cancer, d epression, CHD, and stroke	10
Chen, 2014[37]	Age group, sex, CCI group, stroke types, and a nti-DM drugs	5
Ancelin, 2012[18]	Age, centre, education, baseline cognitive perfo rmance, marital status, BMI, mobility, alcohol, depression, anticholinergic use, chronic respirat ory disorder, hormonal treatment, ischemic pat hologies, HTN, DM, LDL-C, triglyceride, apoE4, and cholesterol exchange transfer protein	19
Bettermann, 2012[8]	Age, sex, race, field center, education, ginkgo biloba randomization group, apoE, stroke, CHD, and use of LLA	10
Beydoun, 201[41]	Age, sex, education, race, smoking, HTN, DM, CAD, dyslipidemia, BMI, systolic blood pressure	11
Parikh, 2011[43]	Age, sex, race, geographic region, insulin, OHA, year in which DM diagnosed, and Hierarchical condition categories	8
Hippisley-Cox, 2010 [42]	Age, sex, BMI, DM, CAD, TCA, SSRI, and depre ssion	7
Li, 2010[13]	Age, sex, education, race, smoking, CASI score s, BMI, apoE, comorbid vascular disease, and o ther LLA	10
Haag, 2009[10]	Age, sex, education, smoking, BMI, systolic blo od pressure, DM, cholesterol, CAD, cerebrovasc	11

	ular disease, and other LLA	
Solomon, 2009[45]	Age, sex, education, blood pressure, BMI, chol esterol	6
Schneider, 2009[44]	Age, race, education, chronic disease burden, c holesterol, NSAID, aspirin	7
Arvanitakis, 2008[19]	Age, sex, Education	3
Cramer, 2008[9]	Education, smoking, DM, apoE, stroke, and 3M S	6
Sparks, 2008[26]	Age, sex, education, apoE	4
Li, 2007[50]	Age at death, sex, CASI, presence of microvasc ular lesions and brain weight	5
Szwast, 2007[49]	Age, sex, education, apoE, and LDL-C	5
Wolozin, 2007[48]	Age, HTN, DM, CAD, and CCI	6
Zigman, 2007[46]	Age, sex, level of MR, BMI, cholesterol, and a poE	6
Rea, 2005[22]	Age, sex, education, alcohol, 3MS, CHD, stroke	7
Zandi, 2005[25]	Age, sex, education, HTN, DM, and apoE	6
Li, 2004[21]	Age, education, apoE, and other LLA	4
Reitz, 2004[23]	Age, sex, education, race, BMI, DM, HTN, hear t disease, and apoE	9
Yaffe, 2002[47]	Age, education, smoking, treatment group, chol esterol, and CABG surgery	6

*Reference number is same with main manuscript.

Abbreviation: Abbreviations: 3MS: Modified mini-mental state examination; AD= Alzheimer's Disease; ApoE= Apolipoprotein E; BMI = Body Mass Index; CABG = Coronary artery bypass graft; CAD = Cardiovascular disease; CASI = Cognitive ability screening instrument; CCI = Charlson comorbidity index; CHD= Coronary Heart Disease; DM = Diabetes; EMR = Electronic Medical Records; HTN= Hypertension; HVLT = Hopkins Verbal Learning Test; LDL-C = Low-density lipoprotein cholesterol; LLA = Lipid lowering agents; MCI = Mild cognitive impairment; MR = Mental retardation; N/A = Not Applicable; NSU: No Statin Use; OHA = Oral hypoglycemic agents; PROCAM = Prospective cardiovascular Munster; SEVLT: Spanish and English

Verbal Learning Test; SSRI = Selective serotonin reuptake inhibitor; SU: Statin User; TCA = tricylic antidepressants.

Supplemental Table S4: Newcastle-Ottawa Quality Assessment Scale (NOS) scores of included studies

Author (year)	Selection	Comparability	Outcome	Total scores
Harding, 2017[35*]	3	2	3	8
Chitnis, 2015[39]	4	2	2	8
Hendrie, 2015[40]	3	2	2	7
Chen, 2014[37]	4	2	2	8
Ancelin, 2012[18]	4	2	2	8
Bettermann, 2012[8]	4	2	2	8
Beydoun, 2011[41]	3	2	2	7
Parikh, 2011[43]	4	2	2	8
Hippisley-Cox, 2010[42]	3	2	2	7
Li, 2010[13]	4	2	3	9
Haag, 2009[10]	4	2	2	8
Solomon, 2009[45]	4	2	2	8
Schneider, 2009[44]	4	1	1	6
Arvanitakis, 2008[19]	3	2	2	7
Cramer, 2008[9]	3	2	3	8
Sparks, 2008[26]	2	2	3	7
Li, 2007[50]	4	2	2	8
Szwast, 2007[49]	4	2	3	9
Wolozin, 2007[48]	4	2	2	8
Zigman, 2007[46]	2	1	1	4
Rea, 2005[22]	4	2	2	8

Zandi, 2005[25]	3	2	3	8
Li, 2004[21]	4	2	3	9
Reitz, 2004[23]	[47]4	2	3	9
Yaffe, 2002[47]	4	2	2	8

*****Reference number is same with main manuscript.

Supplemental Table S5: Random-effects meta-regression analyses of potential moderators of the association of statins use and incident all-caused dementia, Alzheimer's disease and mild cognitive impairment.

<u>**All-caused dementia**</u>

**Clinical variables**	**Number of datasets**	**Slope**<br>**95% CI**	**P-value**	**Intercept**<br>**z**	**P-value**
Age	19	0.002<br>-0.015 to 0.020	0.808	-0.366<br>-0.571	0.568
Male (%)	20	-0.002<br>-0.007 to 0.003	0.406	-0.097<br>-0.604	0.546
Education	8	0.016<br>-0.069 to 0.101	0.709	-0.516<br>-1.004	0.315
**White**	12	**0.005**<br>**0.001 to 0.008**	**0.006**	**-0.533**<br>**-3.617**	**0.001**
Study duration	20	-0.013<br>-0.041 to 0.014	0.334	-0.139<br>-1.258	0.209
Cardiovascular disease (%)	9	0.008<br>-0.001 to 0.017	0.072	**-0.458**<br>**-2.306**	**0.021**
Cerebrovascular disease (%)	10	0.005<br>-0.003 to 0.012	0.216	**-0.290**<br>**-2.346**	**0.019**
DM (%)	14	-0.001<br>-0.005 to 0.003	0.743	-0.115<br>-0.973	0.331
HTN (%)	12	-0.005<br>-0.012 to 0.003	0.233	0.171<br>0.672	0.501
Smoking (%)	6	-0.004<br>-0.023 to 0.015	0.691	-0.221<br>-0.735	0.462
**Cholesterol > 200mg/dl (%)**	5	**0.018**<br>**0.004 to 0.031**	**0.009**	**-1.137**<br>**-2.710**	**0.007**
apoE4 >or=1(%)	10	-0.009	0.357	0.043	0.880

		-0.027 to 0.010		0.150	
BMI>25	7	-0.002<br>-0.029 to 0.025	0.866	-0.139<br>-0.240	0.811
Newcastle total	20	0.170<br>-0.049 to 0.389	0.128	-1.560<br>-1.764	0.078
Number of covariables	20	0.017<br>-0.017 to 0.510	0.322	**-0.361**<br>**-2.269**	**0.023**

Alzheimer`s Disease

**Clinical variables**	**Number of datasets**	**Slope**<br>**95% CI**	**P-value**	**Intercept**<br>**z**	**P-value**
Age	15	0.042<br>-0.003 to 0.088	0.068	**-3.403**<br>**-1.990**	**0.047**
Male (%)	15	-0.003<br>-0.012 to 0.007	0.542	-0.171<br>-0.721	0.471
Education	10	0.008<br>-0.075 to 0.090	0.854	-0.448<br>-0.738	0.461
**White**	10	**0.006**<br>**0.001 to 0.011**	**0.047**	**-0.666**<br>**-2.861**	**0.004**
**Study duration**	14	**-0.063**<br>**-0.121 to -0.005**	**0.033**	0.181<br>0.808	0.419
Cardiovascular disease (%)	9	0.001<br>-0.010 to 0.011	0.884	**-0.395**<br>**-2.279**	**0.023**
Cerebrovascular disease (%)	10	0.001<br>-0.003 to 0.012	0.884	**-0.290**<br>**-2.346**	**0.019**
DM (%)	12	-0.004<br>-0.012 to 0.004	0.304	-0.179<br>-1.015	0.310
HTN (%)	10	-0.007	0.581	0.169	0.808

		-0.032 to 0.018		0.243	
Smoking (%)	6	-0.003<br>-0.025 to 0.020	0.801	-0.404<br>-1.179	0.239
**apoE4 >or=1(%)**	12	**-0.042**<br>**-0.082 to -0.001**	**0.044**	0.858<br>1.585	0.113
BMI>25	6	0.015<br>-0.003 to 0.032	0.094	**-0.811**<br>**-2.207**	**0.027**
Drop-out rate	5	0.005<br>-0.016 to 0.025	0.639	-0.377<br>-1.939	0.053
Newcastle total	15	0.156<br>-0.188 to 0.500	0.375	-1.551<br>-1.098	0.272
Number of covariables	15	0.029<br>-0.009 to 0.067	0.133	**-0.566**<br>**-2.677**	**0.007**

Mild cognitive impairment

**Clinical variables**	**Number of datasets**	**Slope**<br>**95% CI**	**P-value**	**Intercept**<br>**z**	**P-value**
Age	5	-0.009<br>-0.062 to 0.048	0.751	0.556<br>0.293	0.769
**Male (%)**	**6**	**-0.008**<br>**-0.014 to -0.001**	**0.022**	**-0.042**<br>**-2.367**	**0.018**
Study duration	6	0.020<br>-0.017 to 0.058	0.278	-0.219<br>-1.369	0.171
Newcastle total	6	0.317<br>-0.059 to 0.692	0.098	-2.577<br>-1.683	0.092
**Number of covariables**	**6**	**-0.114**<br>**-0.202 to -0.026**	**0.011**	**0.416**<br>**2.282**	**0.023**

Abbreviations: BMI = body mass index; CI = confidence interval; DM = diabetes mellitus; HTN = hypertension; Statistically significant results are in bold

Supplemental Table S6: Checklist of PRISMA guideline.

Section/Topic	#	Checklist Item	Reported on Page #
**TITLE**			
Title	1	Identify the report as a systematic review, meta-analysis, or both.	1
**ABSTRACT**			
Structured summary	2	Provide a structured summary including, as applicable: background; objectives; data sources; study eligibility criteria, participants, and interventions; study appraisal and synthesis methods; results; limitations; conclusions and implications of key findings; systematic review registration number.	5
**INTRODUCTION**			
Rationale	3	Describe the rationale for the review in the context of what is already known.	6-7
Objectives	4	Provide an explicit statement of questions being addressed with reference to participants, interventions, comparisons, outcomes, and study design (PICOS).	7-8
**METHODS**			
Protocol and registration	5	Indicate if a review protocol exists, if and where it can be accessed (e.g., Web address), and, if available, provide registration information including registration number.	21-22
Eligibility criteria	6	Specify study characteristics (e.g., PICOS, length of follow-up) and report characteristics (e.g., years considered, language, publication status) used as criteria for eligibility, giving rationale.	21-22
Information sources	7	Describe all information sources (e.g., databases with dates of coverage, contact with study authors to identify additional studies) in the search and date last searched.	21-22
Search	8	Present full electronic search strategy for at least one database, including any limits used, such that it could be repeated.	21-22

Study selection	9	State the process for selecting studies (i.e., screening, eligibility, included in systematic review, and, if applicable, included in the meta-analysis).	21-22
Data collection process	10	Describe method of data extraction from reports (e.g., piloted forms, independently, in duplicate) and any processes for obtaining and confirming data from investigators.	23-24
Data items	11	List and define all variables for which data were sought (e.g., PICOS, funding sources) and any assumptions and simplifications made.	23-24
Risk of bias in individual studies	12	Describe methods used for assessing risk of bias of individual studies (including specification of whether this was done at the study or outcome level), and how this information is to be used in any data synthesis.	23-24
Summary measures	13	State the principal summary measures (e.g., risk ratio, difference in means).	23-24
Synthesis of results	14	Describe the methods of handling data and combining results of studies, if done, including measures of consistency (e.g., I^2) for each meta-analysis.	23-24
Risk of bias across studies	15	Specify any assessment of risk of bias that may affect the cumulative evidence (e.g., publication bias, selective reporting within studies).	23-24
Additional analyses	16	Describe methods of additional analyses (e.g., sensitivity or subgroup analyses, meta-regression), if done, indicating which were pre-specified.	23-24
**RESULTS**			
Study selection	17	Give numbers of studies screened, assessed for eligibility, and included in the review, with reasons for exclusions at each stage, ideally with a flow diagram.	9-10
Study characteristics	18	For each study, present characteristics for which data were extracted (e.g., study size, PICOS, follow-up period) and provide the citations.	9-10, table 1
Risk of bias within studies	19	Present data on risk of bias of each study and, if available, any outcome level assessment (see item 12).	9-10

Results of individual studies	20	For all outcomes considered (benefits or harms), present, for each study: (a) simple summary data for each intervention group (b) effect estimates and confidence intervals, ideally with a forest plot.	9-13, table 1
Synthesis of results	21	Present the main results of the review. If meta-analyses done, include for each, confidence intervals and measures of consistency.	9-13 figure 2-4
Risk of bias across studies	22	Present results of any assessment of risk of bias across studies (see Item 15).	9-13
Additional analysis	23	Give results of additional analyses, if done (e.g., sensitivity or subgroup analyses, meta-regression [see Item 16]).	10-15, figure 3
**DISCUSSION**			
Summary of evidence	24	Summarize the main findings including the strength of evidence for each main outcome; consider their relevance to key groups (e.g., healthcare providers, users, and policy makers).	14-19
Limitations	25	Discuss limitations at study and outcome level (e.g., risk of bias), and at review-level (e.g., incomplete retrieval of identified research, reporting bias).	18-19
Conclusions	26	Provide a general interpretation of the results in the context of other evidence, and implications for future research.	19
**FUNDING**			
Funding	27	Describe sources of funding for the systematic review and other support (e.g., supply of data); role of funders for the systematic review.	25

From: Moher D, Liberati A, Tetzlaff J, Altman DG, The PRISMA Group (2009). Preferred Reporting Items for Systematic Reviews and Meta-Analyses: The PRISMA Statement. PLoS Med 6(6): e1000097. doi:10.1371/journal.pmed1000097

For more information, visit: **www.prisma-statement.org**.

Supplemental Table S7: search strategy and selection criteria.

Detailed Search Strategy

1) PubMed (through December 27th, 2017)

Keyword: (dementia) AND (statins or statin intervention or statin)

Limited: Human" and "English written"

Results: 632

2) ScienceDirect (through December 27th, 2017)

Keyword: (dementia) AND (statins or statin intervention or statin)

Filter: none

Results: 2766

3) Psychology and Behavior Sciences Collection (from Jan 1st, 1988 to December 27th, 2017)

Keyword: (dementia) AND (statins or statin intervention or statin)

Limited: n/a

Results: 354

4) ClinicalTrials.gov (through December 27th, 2017)

Keyword: dementia AND statin

Limited: n/a

Results: 19

5) Cochrane library (through December 27th, 2017)

Keyword: (dementia) AND (statins or statin intervention or statin)

Limited: n/a

Results: 53

Additional records identified through other sources

Results: 27
